# Multimodal smart suture for dynamic postoperative wound monitoring

**DOI:** 10.1016/j.bioactmat.2026.07.044

**Published:** 2026-07-30

**Authors:** Yang Xiao, Xingyu Gao, Tingting Sun, Jiasheng Jiang, Jing Lu, Chengbin Zhu, Jiaxi Zhang, Yang Sun, Yu Zhang, Xinyi Ke, Gaoshan Huang, Shengda Tian, Xue Ke, Jiajun Li, Zhe Zhao, Xuanyong Liu

**Affiliations:** a Shanghai Engineering Research Center of Nano-Biomaterials and Regenerative Medicine, College of Biological Science and Medical Engineering, Donghua University, Shanghai, 201620, China; b International Institute for Intelligent Nanorobots and Nanosystems, College of Intelligent Robotics and Advanced Manufacturing, and State Key Laboratory of Photovoltaic Science and Technology, Fudan University, Shanghai, 200438, China; c State Key Laboratory of Materials for Integrated Circuits, Shanghai Institute of Microsystem and Information Technology, Chinese Academy of Sciences, Shanghai, 200050, China; d State Key Laboratory of High Performance Ceramics, Shanghai Institute of Ceramics, Chinese Academy of Sciences, Shanghai, 200050, China; e Department of Endocrinology, Seventh People's Hospital of Shanghai University of Traditional Chinese Medicine, Shanghai, 200137, China

**Keywords:** MOF film, Machine learning, Biosensor, Wound monitoring, Thermoelectric

## Abstract

Postoperative wound management remains challenging, owing to the lack of tools for continuous, localized, and quantitative assessment of wound inflammation. To address this limitation, we advance a device-level integration strategy that enables localized multimodal monitoring within a suturable platform. We develop a multifunctional smart suture by engineering a metal-organic framework (MOF) layer onto a conductive thermoelectric (TE) fiber. This unique structure integrates bifunctional electrochemical and thermoelectric sensing on a single platform for real-time monitoring of wound healing dynamics. The precise deposition of metal organic framework layer is fabricated via atomic layer deposition, which provides a highly active interface for sensitive detection of physiologically relevant hydrogen peroxide (H_2_O_2_). This method features ultra-high sensitivity (8450 μA mM^−1^ cm^−2^) and a low detection limit (77.44 nM). Meanwhile, the TE fiber enables accurate local temperature monitoring with a sensitivity of 76.60 μV K^−1^. The smart suture retains sufficient mechanical strength and supports biocompatibility and stable sensing performance for surgical suturing. By fusing multimodal sensing outputs with machine learning-assisted analysis, the system enables classification of inflammation-related states. Evaluated in rat wound models, the suture demonstrates the capability to monitor changes in local inflammatory responses under experimental conditions, highlighting its potential for advanced wound monitoring.

## Highlights

• Smart suture is constructed from ALD-prepared MOF film and thermoelectric fiber for stable inflammation detection.
• Temperature and hydrogen peroxide sensing enable continuous, localized postoperative prognosis.
• Machine learning–based fusion converts multimodal signals into quantitative infection assessment.

## Graphical abstract

**Figure:**
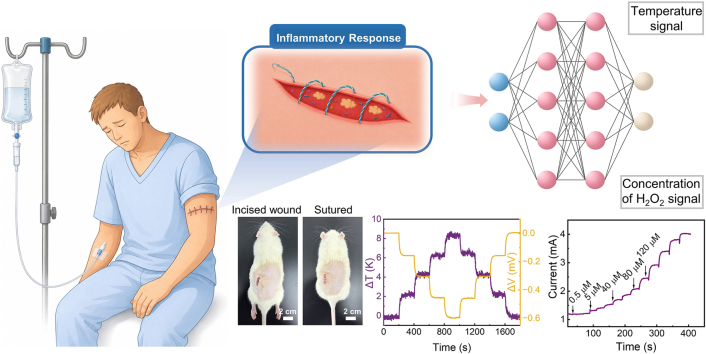

## 1 Introduction

Postoperative wound healing is a highly dynamic process. In current clinical practice, postoperative wound monitoring remains insufficient, primarily due to the lack of continuous and real-time assessment, limited sensitivity to early inflammatory biomarkers, and the restricted range of detectable physiological parameters, which can lead to delayed detection of infection, impaired tissue regeneration, and severe complications [[1], [2], [3]]. Such limitations often result in delayed clinical intervention, as early inflammatory responses are not captured in a timely and quantitative manner. Unlike superficial skin injuries, surgical wounds often present deep and irregular geometries and are subjected to mechanical tension caused by tissue movement [4,5]. Traditional dressings and patch-type sensors, including wound dressings, bandages, and adhesive patches, have been extensively investigated [[6], [7], [8], [9]]. However, these platforms are primarily designed for superficial wounds. They lack conformability to irregular or deep geometries, are prone to detachment in dynamic tissue environments, and cannot provide continuous feedback during the critical postoperative period [10].

Among wound-related indicators, local hydrogen peroxide (H_2_O_2_) concentration and temperature have been recognized as critical biomarkers. During local inflammation, the tissues will produce superoxide through nicotinamide adenine dinucleotide phosphate hydrogen oxidase and quickly convert it into hydrogen peroxide [11,12]. The concentration of hydrogen peroxide will significantly increase. These biomarkers provide important physiological information during healing (e.g., bacterial infection and inflammation) [13,14]. However, relying on a single biomarker often leads to incomplete or even misleading assessment, because postoperative wound healing is a multifactorial process involving tightly coupled biochemical and thermal changes [9]. Therefore, real-time monitoring and evaluation of these biomarkers are essential for effective wound care. However, this need is still not fully addressed in clinical settings [15]. Yet, most current systems are capable of only single-mode sensing, which fails to capture the complexity of the healing postoperative microenvironment and limits accurate evaluation of inflammatory status and tissue regeneration [[16], [17], [18]]. These limitations hinder the accurate assessment of inflammatory status and healing progression. Consequently, there is a pressing need for durable, conformal, and temporally stable multifunctional sensing platforms capable of continuous monitoring throughout the entire postoperative inflammatory and healing phases.

To address these challenges, fiber structures, capable of penetrating deep and geometrically irregular wounds, show great promise for long-term dynamic monitoring [[19], [20], [21], [22]]. However, constrained by challenges in microfabrication techniques compatible with fiber substrates and the coupling of multi-modal sensing signals, most existing fiber-based sutures remain limited to single-mode sensing, which compromises monitoring reliability. More importantly, achieving stable integration of multiple sensing functionalities within a single fiber-based platform remains a significant challenge, particularly in maintaining both sensing performance and mechanical compliance. To address this, we combine a metal–organic framework (MOF)-based electrochemical sensing interface with a carbon nanotube (CNT)-based thermoelectric (TE) fiber, leveraging their complementary properties within a unified system. Notably, thanks to the large surface area, tunable pore structures, and excellent electrochemical properties, MOFs exhibit immense potential serving as excellent H_2_O_2_ sensing materials [23]. Research has revealed that carboxylic acid iron MOFs, constructed from iron (Fe) ions and terephthalic acid, are low in toxicity and biocompatible [[24], [25], [26]]. In addition, owing to the electrical structure, TE fibers possess Seebeck coefficient higher than 40 μV K^−1^ and thereby sensitive temperature response. These complementary properties make MOF and TE fibers suitable for integration within a unified platform, providing a feasible strategy to address the challenge of multimodal sensing integration.

In this study, we develop a smart medical suture by integrating a MIL-53(Fe) sensing layer with a TE fiber via atomic layer deposition (ALD), enabling simultaneous monitoring of H_2_O_2_ and temperature, while machine learning is employed as a supporting tool for multimodal signal fusion and inflammation classification (Figure S1). It should be noted that, unlike prior studies where machine learning is applied to MOF design, synthesis optimization, or property prediction, the machine learning component in this work is used at the device level for multimodal signal fusion and analysis [[27], [28], [29], [30], [31], [32]]. MIL-53(Fe) achieves an exceptional sensitivity toward H_2_O_2_ of 8450 μA mM^−1^ cm^−2^ along with extremely low detection level of 77.44 nM. It also delivers fast response, outstanding stability, and excellent selectivity. Meanwhile, the high Seebeck coefficient of TE fibers endow the suture with high sensitivity to temperature of 76.6 μV K^−1^. More importantly, the fibrous architecture exhibits excellent flexibility, low surface friction, and mechanical compliance with biological tissues, ensuring minimal disruption during suturing, stable fixation at the wound site, and long-term in vivo stability. Considering its practical application as a medical suture, we demonstrated the smart suture by directly applying it to rodent models with either normal or infected incisions. By monitoring changes in H_2_O_2_ levels and temperature at the wound site throughout the healing process, the inflammatory condition can be effectively assessed. This work highlights a device-level integration strategy that enables continuous, multimodal, and localized wound monitoring, demonstrating its potential for intelligent postoperative care in clinically relevant settings.

## 2 Results and discussion

### 2.1 Design of the smart suture for real-time wound monitoring in vivo

**Fig. 1:**
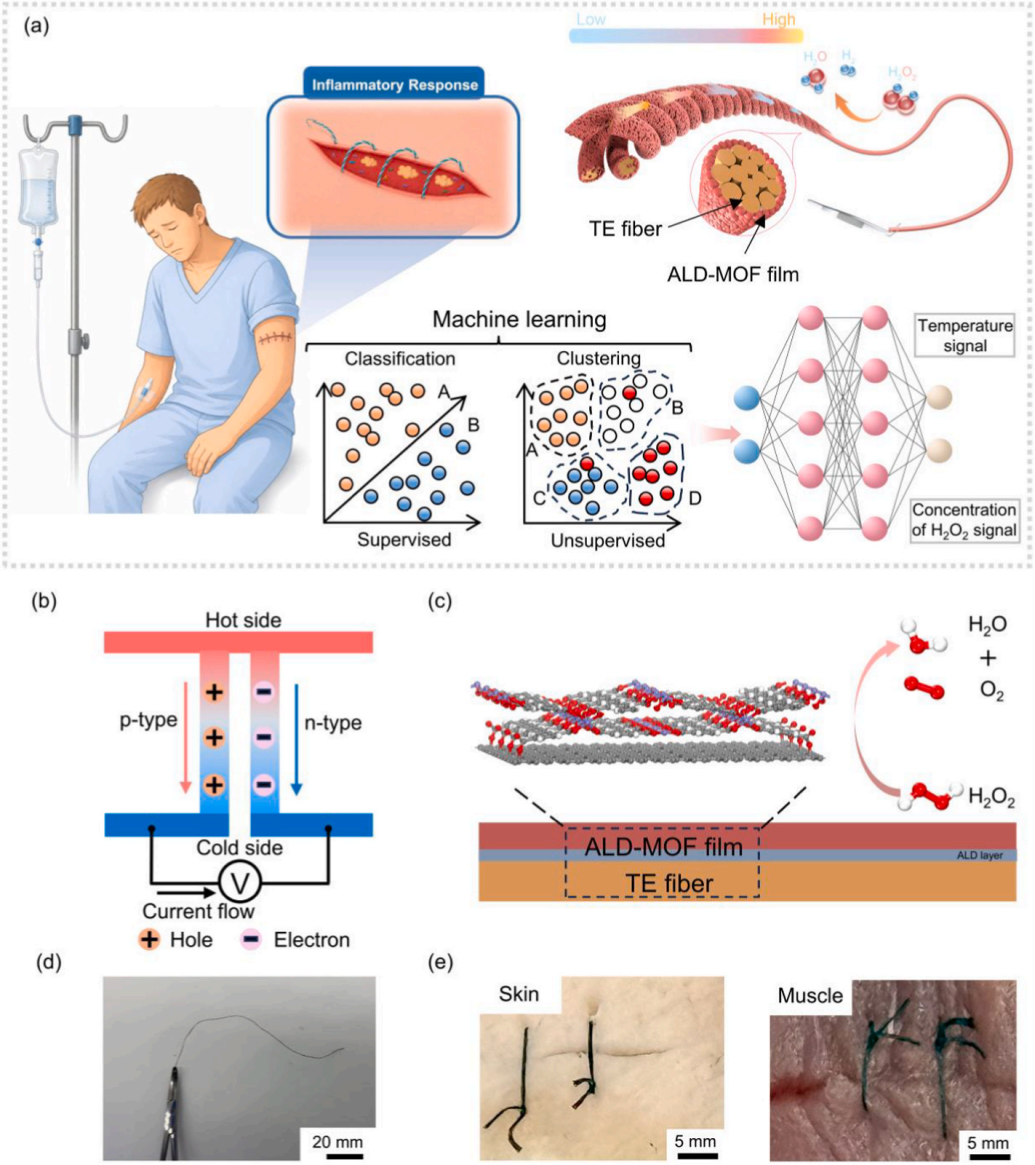
(a) Schematic illustration of the multimodal smart suture for dynamic postoperative wound monitoring. (b) Thermoelectric sensing mechanism of the smart suture. (c) Illustration of the electrochemical sensing on ALD-MOF film. (d) Photograph of the smart suture. (e) Photograph depicting the smart suture sutured onto a fresh porcine skin and muscle.

Unlike conventional surface-based monitoring systems, the fiber-based configuration enables integration into closed or deep wound environments during suturing, allowing localized and in situ sensing within the wound microenvironment throughout the healing process. Fig. 1a illustrates the design concept of a smart suture [33]. It enables the simultaneous monitoring of biochemical (H_2_O_2_) and biophysical (temperature) signals for real-time evaluation of postoperative wound healing at the site. The innovatively designed H_2_O_2_- and temperature-sensing suture serves both as a standard medical suture for the effective closure of surgical wounds and as a continuous monitoring tool, tracking the real-time wound healing progress. To monitor the postoperative wound conditions, the H_2_O_2_ level at the wound site is measured electrochemically using MIL-53(Fe) film sensor [34]. Concurrently, TE fibers function as both the structural backbone of the suture and the active sensing element for temperature measurement. The open voltage of TE fibers demonstrates a linear correlation with temperature difference changes, attributed to their intrinsic Seebeck coefficient [35]. In clinical practice, H_2_O_2_ levels and temperature changes at the wound site are closely associated with wound condition, providing useful indicators of healing status. In particular, under normal conditions, H_2_O_2_ functions as a transient and tightly regulated signalling molecule. However, under pathological inflammatory conditions, it becomes persistently elevated, leading to sustained oxidative stress, tissue damage, and impaired wound healing [13]. Local temperature changes at the wound site reflect inflammatory and metabolic activity. During normal healing, the transient temperature increase is self-limiting and gradually returns to baseline as inflammation resolves. In contrast, under pathological conditions such as infection, activation of inflammatory pathways leads to sustained vasodilation, enhanced metabolic activity, and increased heat generation [3]. Accordingly, the combined monitoring of H_2_O_2_ and temperature is important for a more comprehensive assessment of postoperative wound inflammation. To further leverage the dual sensing capability, the multimodal outputs of temperature and H_2_O_2_ signals were integrated through a machine learning model. This approach enables reliable classification of wound conditions, facilitating interpretation of biosensing data for postoperative monitoring. Fig. 1b and c illustrate the dual-mode sensing mechanism of the smart suture. Fig. 1b illustrates the Seebeck effect, which occurs when there is a temperature gradient at the junction of two different conductors, resulting in an electric potential being generated in the circuit. Fig. 1c shows the uniform growth of an ALD-MOF film on the TE fiber by atomic layer deposition, in which the active sites of the MOF promote the catalytic conversion of H_2_O_2_, enabling electrochemical detection of H_2_O_2_. This design integrates temperature sensing and H_2_O_2_ biochemical sensing into a single smart suture platform. Fig. 1d presents the photographs of the fabricated smart suture, which demonstrates no considerable practical difference with a typical medical suture. In addition, the suture was successfully sutured directly into fresh porcine skin and muscle (Fig. 1e).

The scanning electron microscopy (SEM) (Fig. S2) characterization of the smart suture confirms the homogeneous attachment of the MOF layer to the substrate. And high-magnification SEM images (Fig. S3) reveal closely stacked MOF particles form a continuous film. As shown in Fig. S4, Energy-dispersive X-ray spectroscopy (EDS) mapping analysis verifies the even spread of C, N, Fe, and Zn, reflecting the uniformity of the MOF coating. Fig. S5 shows that the broad coverage of the MOF layer leads to elevated Fe levels. In practical applications, smart sutures require both high electrical conductivity and mechanical stability under deformation, especially in applications where medical sutures undergo intense mechanical deformation during suturing [36,37]. Notably, the TE fiber's more pronounced I–V slope, the smart suture still exhibits reliable conductivity (Fig. S6). Optical microscopy combined with diameter distribution analysis revealed that all three fiber groups exhibited uniform diameter characteristics (Figs. S7–10). Fig. S11 compares tensile strength and ductility of TE fiber, ALD-TE fiber, and smart suture. TE fiber exhibits the highest tensile strength and ductility, whereas tensile strength decreases for ALD-TE fiber and the smart suture. This can be attributed to the highly organized bundle structure and absence of interfacial defects inside the pure TE fiber [38].

In practical applications, maintaining sufficiently low surface friction of the suturing thread is crucial to minimize undesired tissue damage caused by friction during suturing [39,40]. To evaluate the frictional behavior of the smart suture under biologically relevant conditions, the tissue drag force was measured while the suture was pulled through porcine tissue (Fig. S12). The smart suture exhibited a tissue drag force of 0.51 N m^−1^, which is lower than those of commercial silk and polyethylene terephthalate (PET) sutures. This result indicates favorable frictional performance during tissue penetration and suggests the potential to reduce tissue irritation during suturing procedures. The tensile stress–strain curves showed that the smart suture retained acceptable mechanical strength and flexibility after functionalization. Although its tensile strength was lower than that of commercial silk and PET sutures, the smart suture still exhibited sufficient deformability for suturing applications (Fig. S13). To assess the interfacial adhesion between the MIL-53(Fe) film and the TE fibers, the samples were subjected to ultrasonic treatment. As shown in Fig. S14, the MOF film remains uniformly attached to the fiber surface after sonication, without noticeable detachment, demonstrating strong interfacial stability.

### 2.2 Structure characteristics of the smart suture

**Fig. 2:**
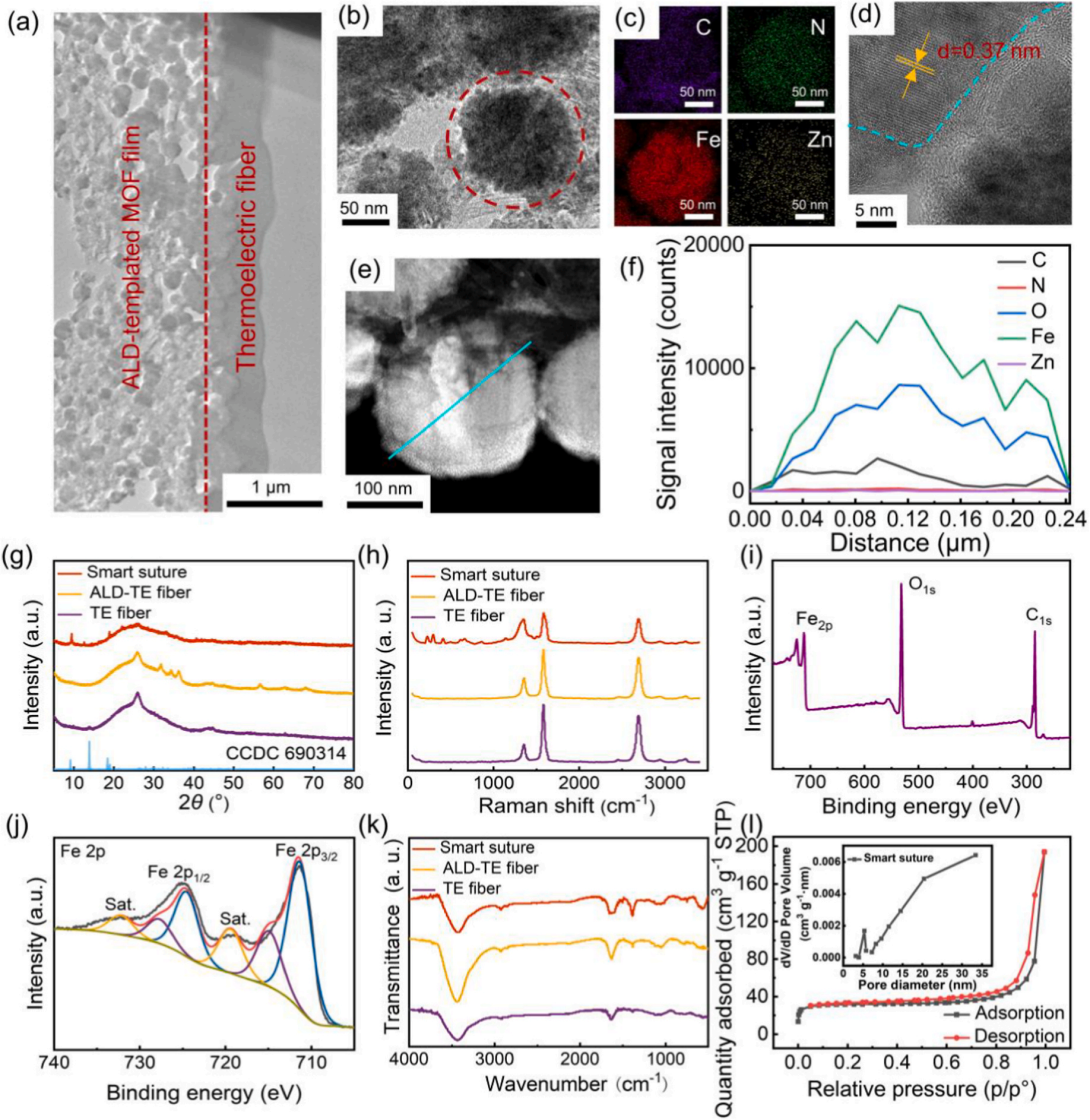
Diagram of the smart suture composite featuring the MOF film. (a) TEM images of the smart suture with low magnifications. (b) TEM image of the smart suture (c) with elemental mapping results. (d) TEM image with high magnification. (e) TEM image of the smart suture with high resolution. (f) The elemental distribution in the smart suture is revealed through an EDS line profile taken along the marked path shown in (e). Structural analysis of the smart suture. (g) XRD patterns of TE fiber, ALD-TE fiber and the smart suture. (h) Raman spectra of TE fiber, ALD-TE fiber and the smart suture. (i) XPS overview spectrum of the smart suture. (j) High-resolution Fe 2p region of the smart suture (k) FTIR spectra of TE fiber, ALD-TE fiber, and the smart suture. (l) The nitrogen adsorption-desorption isotherms for the smart suture are displayed, with pore size distribution shown in the inset.

Fe-based MOFs provide accessible redox-active sites and tunable coordination environments that are favorable for electrochemical H_2_O_2_ detection. To address the intrinsic limitations of particulate MOFs, such as aggregation and limited electrical conductivity, a conformal MOF thin film was constructed on the fiber substrate via ALD-assisted growth. This approach enables uniform coating on the curved fiber surface and improves interfacial stability under mechanical deformation. To examine the structure of the smart suture in greater detail, the cross section of the sample was investigated by focused ion beam-transmission electron microscopy (FIB-TEM), displayed in Fig. 2a. The MOF film uniformly coats the TE fiber, as evidenced by the cross-sectional TEM image. This conformal interface indicates ALD-enabled, precise, and layer-by-layer nucleation on the curved fiber surface, providing intimate contact critical for subsequent electron and ion transport in biosensing [41]. Such a conformal and continuous interface is expected to minimize interfacial resistance and facilitate rapid charge transfer during electrochemical sensing processes. To further evaluate MOF film composition at the nanoscale, Fig. 2b presents a high-resolution TEM image of the MOF-coated region. The image further validates internal uniformity of the nanostructure. The corresponding EDS elemental mappings for C, N, Fe, and Zn demonstrate successful integration of the MOF coating (Fig. 2c). The spatial overlap of these signals also suggests ZnO nanomembrane provides abundant nucleation centers for the development of the MOF film [42]. Notably, Fig. 2d's high-resolution image shows lattice fringes spaced at 0.37 nm, corresponding to the MIL-53(Fe) (311) plane [43]. Fig. 2e presents a radial EDS line scan across an individual MOF particle, while Fig. 2f displays a representative EDS spectrum. Elevated levels of Fe, Zn, C, N, and O indicate the presence of both MOF particles and the oxide nanomembrane. This confirms uniform formation of the MOF layer and retention of its crystallinity upon integration with the fiber, ensuring consistent electronic and structural behavior.

Based on morphological and compositional analyses, we propose a formation mechanism for the MIL-53(Fe) film. It involves a multistep liquid-phase reaction [42]. The initial step entails the generation of an intermediate bimetallic hydroxy double salt (HDS) [43]. During this process, the ALD-deposited ZnO nanomembrane undergoes gradual structural disintegration, allowing Fe^3+^ ions to coordinate with Zn^2+^ species. The resulting flake-like intermediate, composed of [(Zn, Fe_2_) (OH)_2_] Cl_6_, exhibits a characteristic layered structure similar to HDS. This HDS structure resembles that of layered double hydroxides, wherein the main framework consists of cations arranged in layers, with bridging anions facilitating the assembly between inorganic and organic domains [44,45]. This Fe-Zn HDS was synthesized via thermal liquid-phase coordination by introducing Fe^3+^ ions into the ZnO matrix under solvothermal conditions. Upon the subsequent introduction of the organic ligand (H_2_BDC), the protons (H^+^) and hydroxide ions (OH^−^) from H_2_BDC disrupt the HDS crystal structure via ion exchange at active sites [46]. This facilitates transition of the intermediate HDS phase to crystalline MIL-53(Fe) through ligand coordination. Ultimately, this stepwise conversion process enables conformal growth of MOF thin film on TE fiber substrates with high chemical fidelity and structural continuity.

To elucidate the smart suture's structure, X-ray powder diffraction (XRD) patterns of the smart suture, ALD-TE fiber, and TE fiber were acquired, as shown in Fig. 2g. The wide peak apparent between 20 and 30° originates from the carbon substrate, whereas the smart suture exhibits peaks from 10 to 70° that correspond to the MOF layer structure. Notably, the XRD pattern of the smart suture reveals three distinct peaks indexed to the (200), (−110), and (311) planes of MIL-53(Fe), validating its crystalline framework (CCDC 690314). These results further validate the effective formation of an intact MOF coating on the fiber. Additionally, the diffraction pattern for the ALD-TE fiber differs from neat TE fiber, the emergence formation of a distinct new phase. The formation of multicrystalline ZnO nanomembrane prepared via ALD are evidenced by characteristic diffraction peaks at 2*θ* = 31.86, 34.4, 36.16, 47.7, 56.7, and 62.78°, corresponding to the (100), (002), (101), (102), (110), and (103) planes of hexagonal ZnO, respectively [47]. For deeper insight into the fabricated the smart suture, Raman spectra were recorded in the 300-2000 cm^−1^ range, as shown in Fig. 2h. Clearly, a characteristic peak at 1140 cm^−1^ originates from the interaction between sp^2^-hybridized carbon atoms in benzene rings and carboxylate groups. Peaks 1450 and 1500 cm^−1^ correspond to the asymmetric (*γ*_as_ C-O) and symmetric (*γ*_s_ C-O) vibrations of the carboxylate groups (COO^−^) linkers, respectively [48]. Characteristic Raman signals observed near 411, 640, and 864 cm^−1^ are assigned to fingerprint vibrational modes of MIL-53(Fe) [42]. X-ray photoelectron spectroscopy (XPS) was employed to analyze the elemental chemical states in the composite, revealing signals of Fe, O, and C (Fig. 2i). The high-resolution Fe 2p XPS spectrum was deconvoluted into two main peaks at 711.2 eV (Fe2p_3/2_) and 724.6 eV (Fe2p_1/2_), together with satellite features at 719.4 and 732.3 eV, which are characteristic of Fe(III) species [49]. The preserved Fe(III)-based coordination environment provides abundant accessible redox-active centers that are favorable for catalytic H_2_O_2_ sensing (Fig. 2j) [50].

FTIR spectroscopy was performed to investigate the chemical structure evolution during the fabrication of the smart suture. Fig. 2k shows the FTIR spectrum of the smart suture with several well-defined peaks at approximately 1600 cm^−1^ and 1390 cm^−1^. These signals are attributed to the symmetric and asymmetric stretching vibrations of coordinated –COO^-^ groups [51]. Compared to TE fibers and ALD-TE fibers, the presence of a distinct absorption band around 540-600 cm^−1^ corresponds to the Fe–O stretching vibration in smart sutures, further confirming the formation of iron-carboxylate coordination bonds [52]. Furthermore, nitrogen adsorption–desorption isotherms were obtained to assess the porous characteristics and surface area, as shown in Fig. 2l and inset. The smart suture displays a typical type IV isotherm with clear hysteresis, suggesting a well-developed mesoporous structure, with a Brunauer–Emmett–Teller (BET) surface area of the smart suture is 118 m^2^ g^−1^ [53]. This result confirms the high porosity and effective surface coverage of the MOF films. This value reflects the composite nature of the MOF-coated fiber system. Notably, the presence of a hierarchical mesoporous structure facilitates efficient mass transport and electrolyte diffusion, while the conformal thin-film configuration enables effective exposure of electroactive sites across the fiber surface. Such structural features are critical for electrochemical sensing performance.

### 2.3 Sensor characterization of the smart suture

**Fig. 3:**
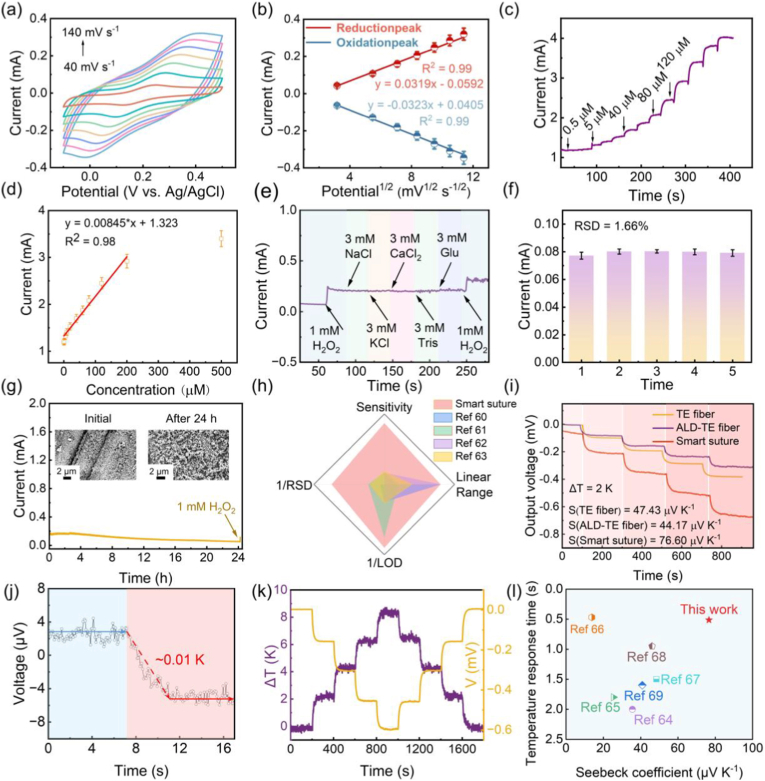
Performance evaluation of the smart suture featuring MIL-53(Fe) film: electrochemical and thermoelectric aspects. (a) CV measurements were conducted using a 5 mM K_3_[Fe(CN)_6_] solution. (b) Reduction (red) and oxidation (blue) peak responses are plotted versus the square root of the scan rate based on CV results. (c) Real-time current response of the smart suture with addition of H_2_O_2_ at 1.2 V. (d) Current responses of the smart suture as functions of H_2_O_2_ concentration. (e) *I-t* curve response of the smart suture with 1 mM H_2_O_2_, 3 mM NaCl, KCl, CaCl_2_, Tris, Glu, and 1 mM H_2_O_2_ added. (f) Statistical analysis of the smart suture electrode responses to five successive additions of 1 mM H_2_O_2_. (g) *I-t* curve response of the smart suture for a 24 h stability test with 1 mM H_2_O_2_ at 1.2 V. SEM images of the sample before and after the test are shown in the insets. (h) Comparison of sensing performance, including relative sensitivity, relative standard deviation (RSD), limit of detection (LOD), and linear range, between the smart suture and other sensors reported in the literature. Ref [54]: Laser-induced graphene (LIG) surface modified with Prussian blue (iron hexacyanoferrate); Ref [55]: Na, O-co-doped-graphitic-carbon nitride (Na,O-g-C_3_N_4_); Ref [56]: chitosan/polyaniline grafted graphene oxide nanohybrid composite coating (CS/GOP); Ref [57]: AuNPs were posited onto porous Gallium nitride (AuNPs/porous GaN) [[54], [55], [56], [57]]. (i) The output voltages of TE fiber, ALD-TE fiber, and the smart suture at various steady temperature differences. (j) The smart suture exhibits an ultrahigh temperature sensitivity, with a minimum detectable temperature difference as low as 0.01 K. (k) Reversible thermoelectric response of the smart suture under cyclic temperature loading. (l) Comparison of the temperature response time and Seebeck coefficient of this work with other reported studies. Ref [58]: microstructure-frame-supported organic thermoelectric (MFSOTE); Ref [59]: titanium carbide (MXene)-silver nanowire (AgNW)-PEDOT:PSS-tellurium nanowire (TeNW) nanocomposite (MXene-AgNW/PEDOT:PSS-TeNW); Ref [60]: poly(3,4-ethylenedioxythiophene)/multi-walled carbon nanotube (PEDOT/MWCNT); Ref [61]: polydopamine is used as a linker to connect carbon nanotubes with carbonized glucose, forming a protective layer on the carbon nanotube surfaces (c-SWCNT/PDA/Glu); Ref [62]: MOF/carbon nanotube (CNT) thermoelectric hybrids (MOF/CNT); Ref [63]: composite based on single-walled carbon nanotubes (SWCNT) and biodegradable polycaprolactone (PCL) (PCL-SWCNT) [[58], [59], [60], [61], [62], [63]].

To quantitatively analyze the electrochemical activity, K_3_[Fe(CN)_6_] was used as a probe to determine the active area of the smart suture. As depicted in Fig. 3a and b, CV profiles of the smart suture electrode exhibit well-defined redox peaks. The corresponding peak currents demonstrate a linear dependence on scan rates within 40–140 mV s^−1^, characteristic of surface-confined electrochemical processes. The Randles-Sevcik equation was then applied to calculate the active area (*S*) of the electrode material [64]:

*I*_peak_ = (2.69 × 10^5^)*n*^3/2^*SD*^1/2^*Cv*^1/2^

where *n* represents the number of transferred electrons, *D* is the diffusion coefficient of K_3_[Fe(CN)_6_], and *C* denotes the bulk concentration of K_3_[Fe(CN)_6_]. Here, the *S* was calculated to be 0.00924 cm^2^ from the slope of the peak current versus the square root of the scan rate.

The electrochemical analysis of the smart suture electrode using cyclic voltammetry (CV) in 1 mM H_2_O_2_ reveals redox peaks at 0.5 V (oxidation) and 0.3 V (reduction), as illustrated in Fig. S15. These findings suggest the electrode has the capacity to catalyze oxidation of H_2_O_2_, leading to the generation of additional current. Additionally, we evaluated the electrochemical response by analyzing current-time (*I-t*) curves to determine the optimal potential for H_2_O_2_ detection. The electrocatalytic performance of the smart suture sensor was systematically evaluated at different applied potentials (0.9, 1.2, and 1.5 V) in 0.1 M PBS (Phosphate Buffered Saline). As illustrated in Fig. S16, the sensor exhibited a significantly enhanced current response toward H_2_O_2_ at 1.2 V, indicating optimal sensing performance under this condition. Fig. 3c further shows the *I–t* response at 1.2 V of the smart suture electrochemical sensor upon stepwise introduction of various H_2_O_2_ concentrations. When H_2_O_2_ is added, a clear and rapid current response is observed, demonstrating excellent instantaneous sensing performance. Fig. 3d illustrates calibration (concentration–response) plots obtained from analysis of three data sets corresponding to different H_2_O_2_ concentrations. Based on the calibration curve, the smart suture shows a remarkable sensitivity of 8450 μA mM^−1^ cm^−2^ with a linear range from 0.5 to 200 μM (linear regression equation: Y = 0.00845X + 1.323; correlation coefficient (R^2^) = 0.98). The limit of detection (LOD) is determined by using the formula: LOD = 3δ/S [65], where *δ* represents the standard deviation and *S* is the sensitivity, resulting in a LOD of 77.44 nM. Furthermore, we evaluated the sensor functionality of the samples containing and excluding the MOF film. Fig. S17 illustrates that the smart suture exhibits a clear response to H_2_O_2_, whereas pure TE fiber shows nearly no sensing performance. Thus, the sensor functionality originates from the MOF layers on the TE fibers.

The selectivity capability is a crucial factor in evaluating the performance of the sensor. Fig. 3e shows the selectivity of the smart suture. The electrochemical response of the smart suture was assessed by sequentially introducing 1 mM H_2_O_2_ and 3 mM of common interfering species typically presented in wound exudates, including NaCl, KCl, CaCl_2_, Tris, and glucose (Glu) [66]. A significant current response is observed exclusively upon the addition of H_2_O_2_, while the presence of other species elicits negligible interference, confirming the sensor's high selectivity toward H_2_O_2_. To further investigate the sensor's susceptibility to trace levels of interferents, an additional test was conducted using lower concentrations of interferents (0.1 mM), as illustrated in Fig. S18. The *I–t* curves upon stepwise introduction of H_2_O_2_ (1 mM), interfering substance (0.1 mM NaCl, KCl, CaCl_2_, Tris and Glu) exhibit negligible current changes due to the interferents, demonstrating the sensor's strong anti-interference performance. Furthermore, the antifouling capability of the smart suture was evaluated by comparing the current responses toward successive H_2_O_2_ additions in PBS with and without bovine serum albumin (BSA). As shown in Fig. S19, after incubation in BSA for 5 min, the smart suture retained nearly unchanged stepwise current responses, indicating negligible protein interference during H_2_O_2_ detection. To further evaluate sensing performance in biologically relevant environments, H_2_O_2_ detection was performed in simulated body fluid (SBF), fetal bovine serum (FBS), 10% FBS, and 10% heat-inactivated FBS. As shown in Fig. S20, distinct stepwise current responses were observed in all media, demonstrating the reliable sensing performance of the smart suture under complex biological conditions.

The sensor's high repeatability is demonstrated by five *I-t* curves toward H_2_O_2_ obtained from the same smart suture (Fig. S21), with a relative standard deviation (RSD) of 2.23% (Fig. 3f). In addition, the batch-to-batch consistency of the smart suture was evaluated to assess the reproducibility of the fabrication process. Ten batches of smart sutures prepared from independent synthesis systems were tested under identical conditions. After the addition of 1 mM H_2_O_2_, all samples exhibited comparable current responses (Fig. S22), indicating good fabrication reproducibility and consistency. The electrochemical sensor's stability was further assessed, and Fig. 3g illustrates that the composite maintains excellent stability with a consistent current response over 24 h. The structural stability of the smart suture is confirmed by the SEM images (insets of Fig. 3g) taken before and after the stability test. To further evaluate the dynamic response capability of the smart suture, randomly ordered additions of H_2_O_2_ at alternating concentrations (0.5 and 80 μM) were performed (Fig. S23). The smart suture generated distinct current responses at both low and high concentrations.

During the wound healing process, the pH value of the local microenvironment undergoes dynamic changes. By continuously adding 1 mM H_2_O_2_ under different pH conditions, the smart suture exhibited a stable and significant stepped current response. The results indicate that this biosensor can withstand the pH fluctuations (ranging from 5 to 8) during the wound healing process and reliably detect H_2_O_2_ (Fig. S24). To further evaluate the mechanical reliability of the smart suture under practical use conditions, representative mechanical deformations, including bending, winding, and friction, were investigated. As shown in Fig. S25, the smart suture maintained stable structural integrity under bending deformation and could withstand an external load of 20 g, indicating good flexibility and mechanical robustness. Furthermore, the current responses remained relatively stable under bending angles of 0−120° (Fig. S26a), after 150 bending cycles (Fig. S26b), and following 60 friction cycles (Fig. S26c). These results indicate that the smart suture can maintain stable sensing performance under typical mechanical deformation and friction conditions relevant to practical applications. The conformal MOF thin film, integrated with the conductive CNT fiber substrate, facilitates efficient charge transfer and stable interfacial contact. This architecture enables effective utilization of the MOF active sites, contributing to the enhanced sensitivity and selectivity of the smart suture compared to systems based on particulate MOFs or conventional electrodes [67,68]. This hybrid structure is therefore expected to enhance the sensitivity, selectivity, and stability of sensing systems [69]. As a result, the smart suture exhibits superior sensing performance compared to MOF particles and other electrode materials (Fig. 3h and Table S1) [[54], [55], [56], [57]].

The sensing performance of the smart suture was further evaluated. We first conducted dynamic thermal response measurements under varying temperature differences (ΔT) to reveal the temperature sensing ability. As shown in Fig. 3i, the suture exhibits an immediate and stable thermoelectric voltage (ΔV) response to stepwise temperature changes, reflecting rapid response. And as displayed in Fig. 3i and Figs. S27–S29, all the TE fiber, ALD-TE fiber, and the smart suture show stepwise and highly linear output voltage profiles across incremental temperature stimuli. More importantly, among these fibers, the smart suture shows the highest voltage response under identical temperature gradients, attributing to synergistic effects of MOF film and CNT substrates. And the Seebeck coefficient (α) of the smart suture is calculated to be 76.6 μV K^−1^, i.e. the thermal sensitivity is 76.6 μV K^−1^, whose value is higher than that of the TE fiber and ALD-TE fiber, reflecting ultra-responsive feature. The close tracking of ΔV to ΔT in real time suggests the smart suture can effectively capture subtle temperature fluctuations under physiological conditions. This capability is essential for monitoring inflammatory progression and postoperative wound healing stages.

To systematically investigate the thermoelectric sensing performance of the smart suture for postoperative wound monitoring, its dynamic response was examined. As shown in Fig. 3j and Fig. S30, a temperature difference of 0.01 K was initially established (point A), and a thermoelectric voltage response was subsequently generated after 514 ms (point B), producing a voltage of approximately 8 μV. As shown in Fig. S31, with thermoelectric voltage synchronously decreasing as ΔT increased. This behavior demonstrates the high sensitivity and rapid responsiveness of the smart suture under thermal stimuli. To further assess long-term reliability, cyclic heating–cooling tests were performed (Fig. 3k). During repeated loading and unloading, the ΔV traces were nearly identical with negligible hysteresis, confirming the excellent reversibility and operational stability of the thermoelectric response, which is essential for reliable temperature sensing in wound healing applications. When compared to the latest thermoelectric-based temperature sensors, smart suture exhibits a rapid temperature response of up to 514 ms and Seebeck coefficient of 76.6 μV K^−1^, both of which represent the high-performance levels in available thermoelectric devices compared with previous reports (Fig. 3l and Table S2) [[58], [59], [60], [61], [62], [63]]. To further evaluate the robustness of the smart suture under clinically relevant suturing conditions, repeated penetration tests were performed using fresh porcine skin. After 30 consecutive penetration cycles, SEM images revealed that the MOF coating remained continuous without obvious cracking or delamination, while EDS elemental mapping confirmed that the distributions of Fe and Zn remained uniform (Figs. S32 and S33). Furthermore, the electrochemical response toward H_2_O_2_ and the thermoelectric response exhibited negligible changes after repeated tissue penetration (Fig. S34), demonstrating excellent coating stability and sensing durability under practical mechanical loading. To further evaluate the overall performance and application potential of the proposed smart suture platform, representative fiber-based and implantable sensing systems reported in previous studies were comparatively summarized in Table S3 [[70], [71], [72], [73]]. The comparison highlights the capability of the present smart suture system for multimodal sensing and localized monitoring within complex physiological environments.

### 2.4 Mechanism of enhanced sensing

**Fig. 4:**
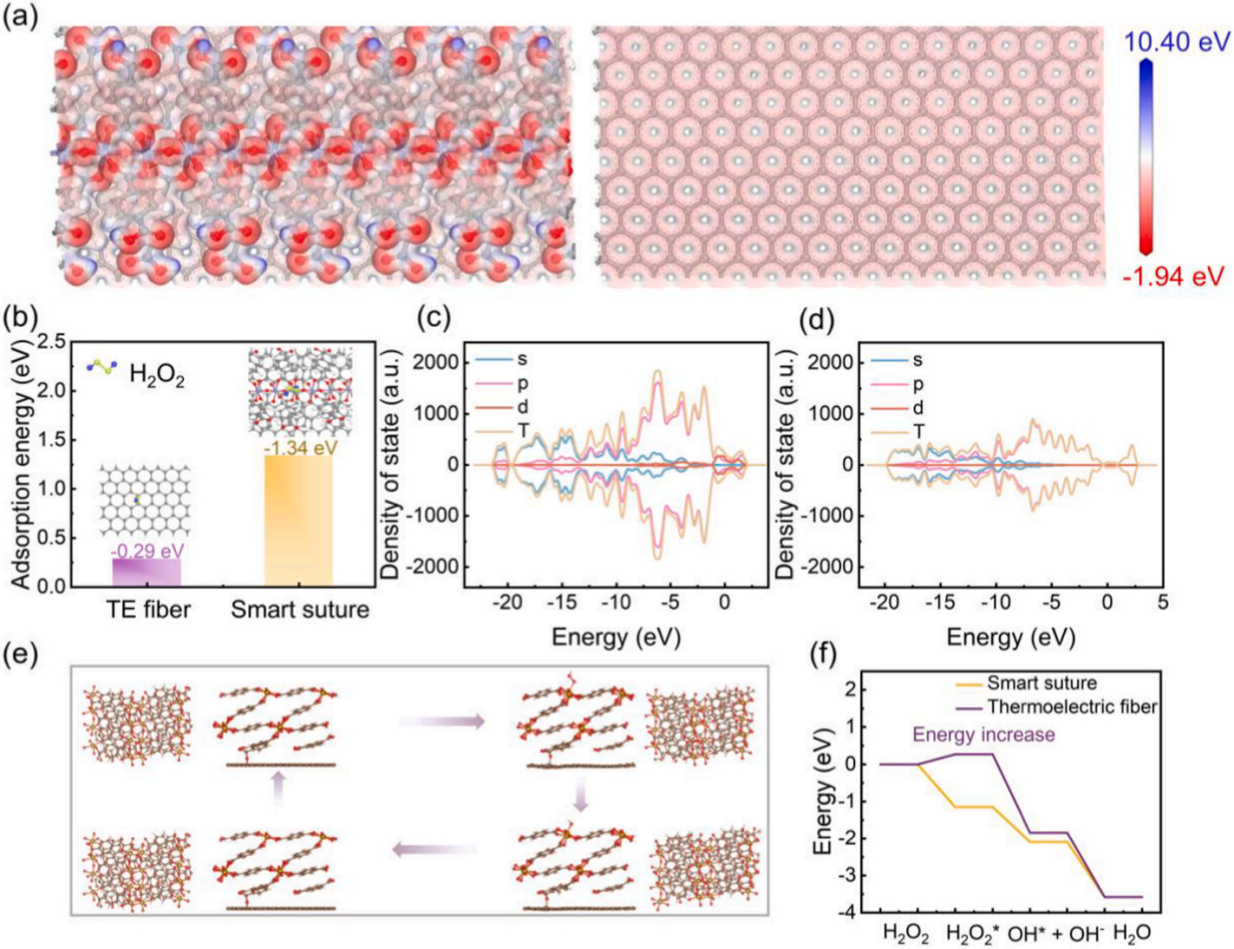
Sensing mechanism of smart sutures. (a) Electrostatic potential on TE fiber and smart suture. (b) Corresponding adsorption energy of H_2_O_2_ on TE fiber and smart suture. Projected Density of States (PDOS) of (c) smart suture and (d) TE fiber. (e) H_2_O_2_ reaction routes on smart suture (top and side view). (f) Energy diagrams depicting the free energy variations during the proposed reactions.

To further elucidate the exceptional sensing performance of the smart suture, first-principles calculations were performed to examine its adsorption behaviour towards H_2_O_2_ molecules, electronic structure, and catalytic pathways. As shown in Fig. 4a, the electrostatic potential distribution reveals that the smart suture exhibits a more heterogeneous potential landscape than the TE fiber. The Fe sites in MIL-53(Fe) markedly enhance the local electrostatic field, promoting charge redistribution and strengthening the adsorption affinity for H_2_O_2_ molecules. Adsorption energy calculations (Fig. 4b) reveal that H_2_O_2_ adsorption on the smart suture (−1.34 eV) is much stronger than on TE fibers (−0.29 eV), indicating that the MOF domains provide stronger active binding sites, stabilise H_2_O_2_ intermediates, and promote their catalytic decomposition. This finding is further supported by electronic structure analysis. PDOS analysis (Fig. 4c and d) demonstrates that the smart suture exhibits pronounced orbital hybridisation near the Fermi level, which enables efficient charge transfer to H_2_O_2_, whereas TE fibers display only weak hybridisation. Such orbital coupling accelerates charge transfer within H_2_O_2_ molecules, whereas the weaker orbital contributions of TE fibers limit their catalytic activity.

To better understand the sensing mechanism, theoretical simulations were performed to examine the catalytic effects of MOF-coated smart sutures. Fig. 4e and S35, S36 show the reaction pathways of H_2_O_2_ on the surfaces of the smart suture and TE fiber during the sensing process. Briefly, H_2_O_2_ first adsorbs onto Fe active sites, followed by dissociation into hydroxyl groups. Those hydroxyl groups remain adsorbed on active sites. Subsequently, one hydroxyl group desorbs from Fe active site. A proton reacts with the remaining hydroxyl group to form H_2_O, which remains adsorbed on the active site. Upon desorption, the catalyst surface is restored to its original form [74]. The overall reaction pathway on TE fiber is similar to that on the smart suture. The smart suture, constructed by growing a MIL-53(Fe) thin film on a TE fiber substrate, provides a highly porous structure with abundant Fe-based catalytic sites, facilitating efficient H_2_O_2_ adsorption and subsequent decomposition. In contrast, bare TE fiber lacks these active metal centers, resulting in limited adsorption and a higher activation barrier for H_2_O_2_ dissociation. The smart suture demonstrates a dense and interconnected network of Fe coordination sites that enhance electron transfer and catalytic efficiency. TE fiber, on the other hand, presents a relatively inert and smooth carbon lattice.

This difference is quantitatively reflected in free energy diagrams (Fig. 4f). Regarding the catalytic reaction producing reactive hydroxyl radicals from H_2_O_2_: H_2_O_2_→H_2_O_2_*→OH* + OH^−^, pure TE fiber absorbs 0.27 eV externally, while the smart suture releases 1.15 eV externally. These results demonstrate that the smart suture's catalytic capability is significantly greater than that of pure TE fiber. The MIL-53(Fe) film reduces the activation energy of H_2_O_2_ activation, thereby facilitating the reaction. Overall, simulations indicate that the MIL-53(Fe) film offers numerous active sites enabling efficient catalysis. Simulations also indicate that the MIL-53(Fe) film significantly reduces energy barrier for H_2_O_2_ decomposition, facilitating a more favorable electron transfer pathway. Therefore, careful design of MOF films can significantly improve sensing capabilities.

### 2.5 Proof-of-concept validation of Bayesian-based multimodal signal fusion

**Fig. 5:**
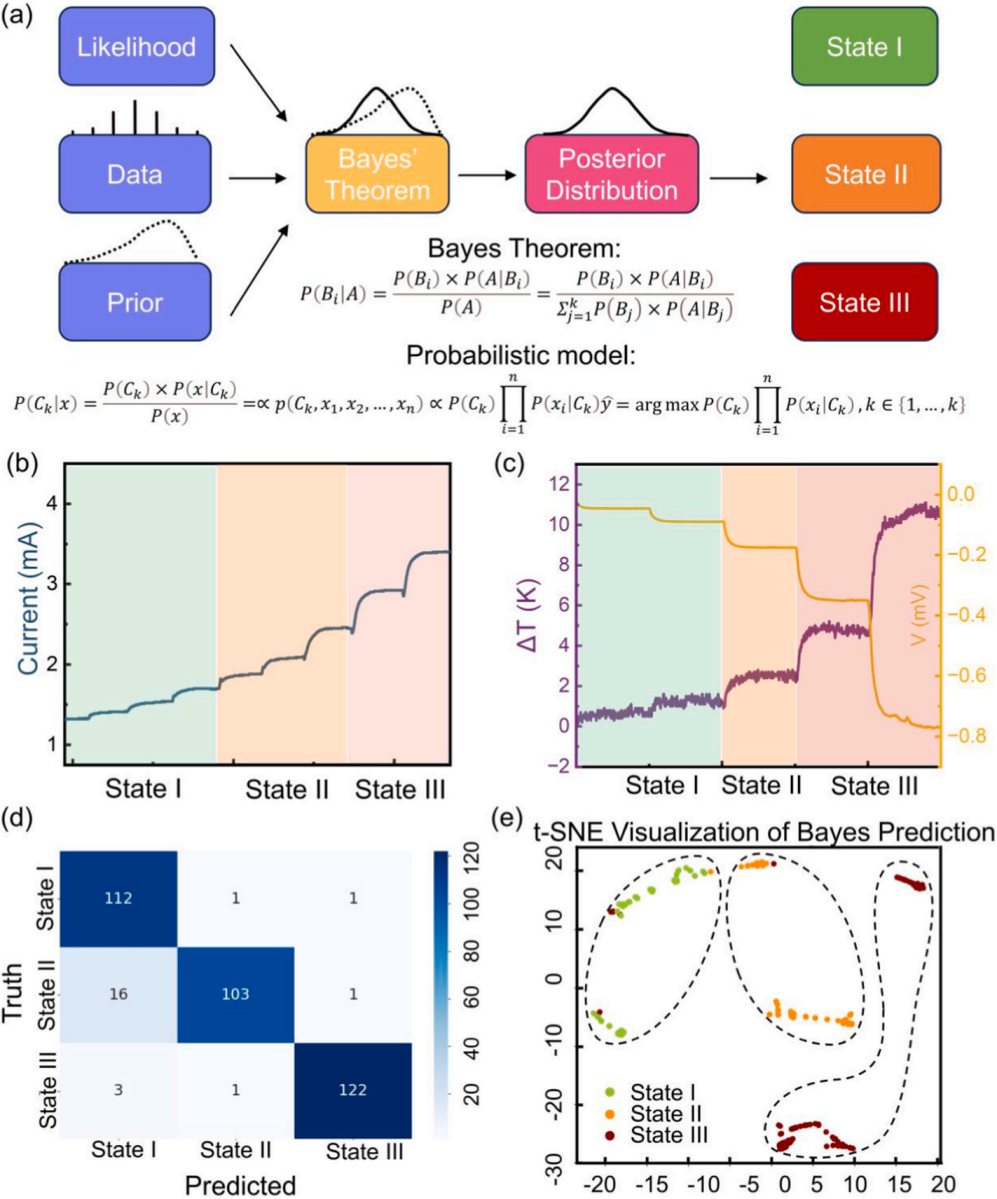
Bayesian inference-based classification of three predefined inflammation-associated sensing states (State I–III, corresponding to mild-, moderate-, and severe-like inflammatory conditions). (a) Schematic of the Bayesian probabilistic model [78]. (b) Current responses to different H_2_O_2_ concentrations representing three predefined sensing states. (c) Time-dependent thermoelectric responses of the smart suture under stepwise temperature gradients corresponding to the three predefined sensing states. These predefined sensing states were established under controlled in vitro conditions for proof-of-concept evaluation of Bayesian-based multimodal signal fusion and do not represent definitive clinical diagnostic grades. (d) Confusion matrix showing classification accuracy. (e) t-SNE visualization of Bayesian prediction results.

To further analyze the multimodal sensing data generated by the smart suture, Bayesian inference was employed as a supporting tool for signal fusion and inflammation classification. The objective of this analysis was to evaluate the feasibility of multimodal signal fusion under representative sensing conditions before in vivo validation. It should be noted that the machine learning component in this work is not used for MOF design, synthesis optimization, or property prediction, but is applied at the device level for multimodal signal fusion and analysis. Recent studies have demonstrated the growing potential of artificial intelligence in biomedical applications, including AI-assisted wound-state assessment through multiplexed sensing platforms, as well as optimization of wound-healing materials and biofabrication processes [75,76]. For example, an AI-enabled multiplexed sensor patch has been reported for wound monitoring and classification based on multiple physiological indicators, highlighting the value of intelligent analysis for wound-state evaluation [77]. As shown in Fig. 5a, the classifier was developed under the framework of Bayesian decision theory, which selects the class label by minimizing the expected conditional risk and enables efficient probabilistic classification. To simplify the computational process, a naïve Bayes assumption was adopted, where sensing features were treated as conditionally independent without substantially compromising classification performance [78]. Rather than establishing clinically validated inflammation grades, three representative signal-associated wound states (State I–III) were operationally established under predefined H_2_O_2_ concentrations and temperature conditions to simulate mild-, moderate-, and severe-like inflammatory microenvironments. These predefined states were introduced solely for proof-of-concept evaluation of multimodal signal fusion and Bayesian classification [3,79]. Fig. 5b presents the electrochemical responses of the smart suture to different H_2_O_2_ concentrations, while Fig. 5c shows the corresponding thermoelectric responses under different temperature gradients. These predefined sensing conditions were established to simulate representative wound microenvironments for proof-of-concept evaluation of Bayesian-based multimodal signal fusion. By integrating biochemical and thermal sensing information, the multimodal platform enabled probabilistic characterization of dynamic wound inflammatory conditions. Compared to chemical signals, the thermoelectric response offers a complementary physical modality, thereby reducing uncertainty from single-signal fluctuations. Next, 80% of the data were used for training and the remaining 20% for testing. Fig. 5d shows that the Bayesian classifier accurately distinguished the three predefined signal-associated wound states. The y-axis represents the actual grades, while the x-axis denotes the predicted grades. The classification confusion matrix demonstrates a recognition accuracy of 94% with very few misclassifications among the three categories. The T-distributed stochastic neighbor embedding (t-SNE) visualization (Fig. 5e) reveals clear clustering of clustering of the three predefined wound-state categories. The quantitative results from the confusion matrix and the intuitive separation from t-SNE mutually corroborate the robustness of the classification. Overall, these results demonstrate the feasibility of integrating electrochemical and thermoelectric signals through Bayesian inference for multimodal wound-state assessment.

### 2.6 In vivo demonstration of the smart suture

**Fig. 6:**
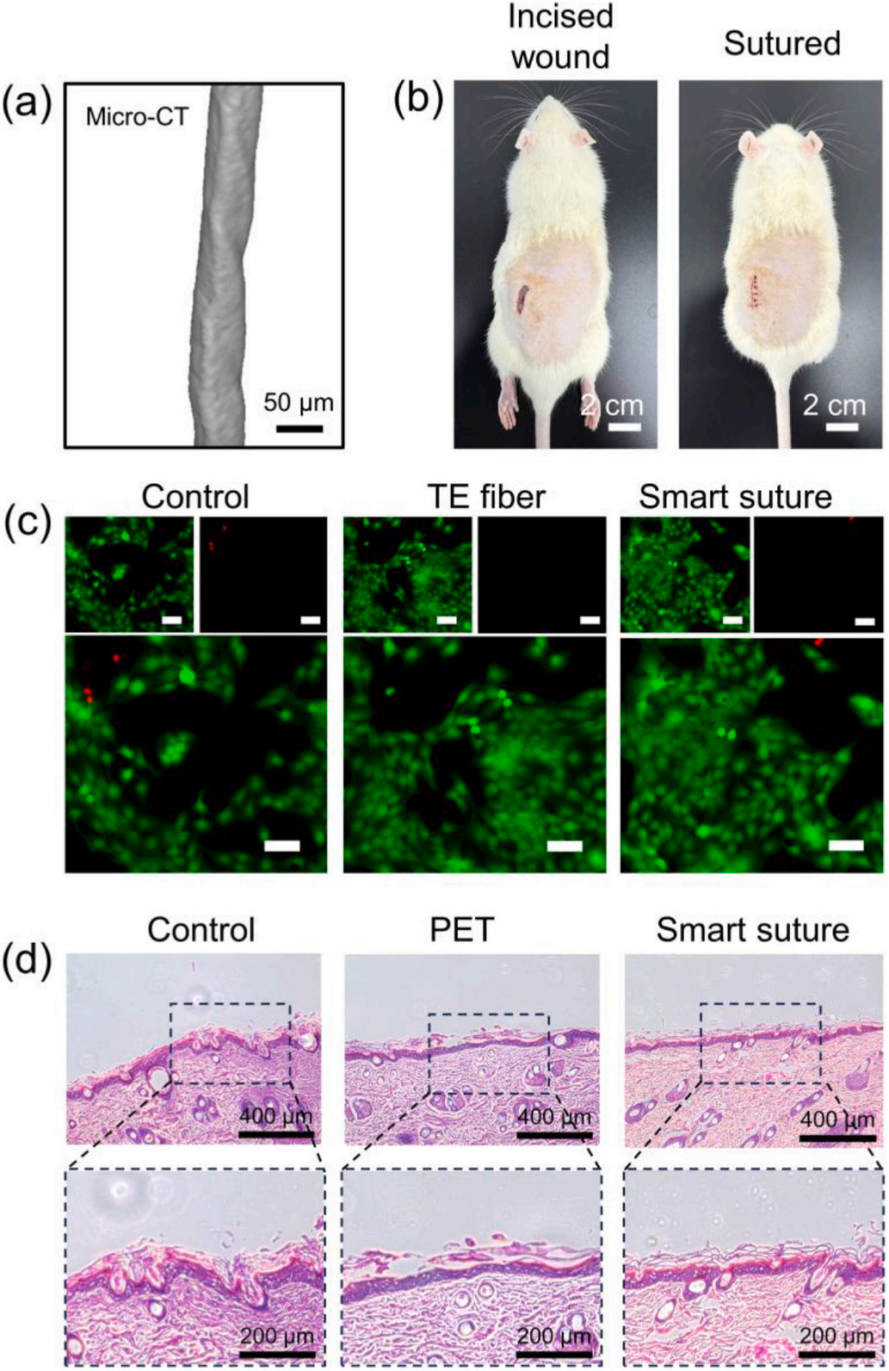
In vivo application and biocompatibility evaluation of the smart suture. (a) CT modelling images of the smart suture. (b) Photograph of a rat before (left) and after (right) the application of the smart suture on an incised wound on the dorsal skin. (c) Biocompatibility of the smart suture (scale bar: 20 μm). (d) Representative images of skin wound tissue specimens stained with H&E.

As shown by micro-CT depicted in Fig. 6a, the smart suture exhibits structural integrity and uniformity. Fig. 6b presents representative photos of the rat's back skin wound before and after suturing. To verify the feasibility of the smart suture for postoperative wound monitoring, we first conducted a biocompatibility test. The biocompatibility of the smart suture was evaluated using human oral keratinocytes (HOK) cells. These cells were co-cultured with either the smart suture or its constituent fiber. Fig. 6c shows fluorescent images from live/dead staining after up to 72 h of culture. Negligible cytotoxicity is observed in all tested groups compared to control group cultured on a standard cell culture plate. This demonstrates the excellent biocompatibility of the smart suture. Subsequently, commercial and smart sutures were implanted into the dorsal skin of rat for 7 days. The cumulative release of Fe was then evaluated in intact skin as well as in tissues treated with commercial and smart sutures. Iron-carboxylate MOFs, including MIL-53(Fe), have been reported to exhibit controlled degradation and gradual release of iron-containing species under aqueous or mildly acidic biological conditions through partial dissociation of metal–carboxylate coordination bonds. Such controllable degradation behavior has been explored in biomedical systems for controlled delivery applications [80,81]. The Fe concentration released from the smart suture (∼1.02 ppm) is slightly higher than that of the control and commercial groups, but remains well below reported cytotoxic thresholds for iron ions [82,83]. These results indicate that the Fe release occurs at a low level within a biologically acceptable range over the 7 days period (Fig. S37). To further evaluate the in vivo biosafety and tissue response of the implanted smart suture, histological analyses were subsequently performed. As shown in Fig. 6d–H&E-stained histological sections obtained on day 16 revealed intact skin tissue architecture with negligible inflammatory cell infiltration in the control, TE fiber, and smart suture groups, indicating favorable in vivo biocompatibility of the implanted materials. The results collectively demonstrate that the smart suture possesses good in vivo biocompatibility and does not induce excessive inflammatory responses during the implantation period. After implantation, the smart sutures closely conformed to the wound area without causing significant tissue damage or abnormal inflammatory responses, confirming their suitability for in vivo wound closure and monitoring applications.

**Fig. 7:**
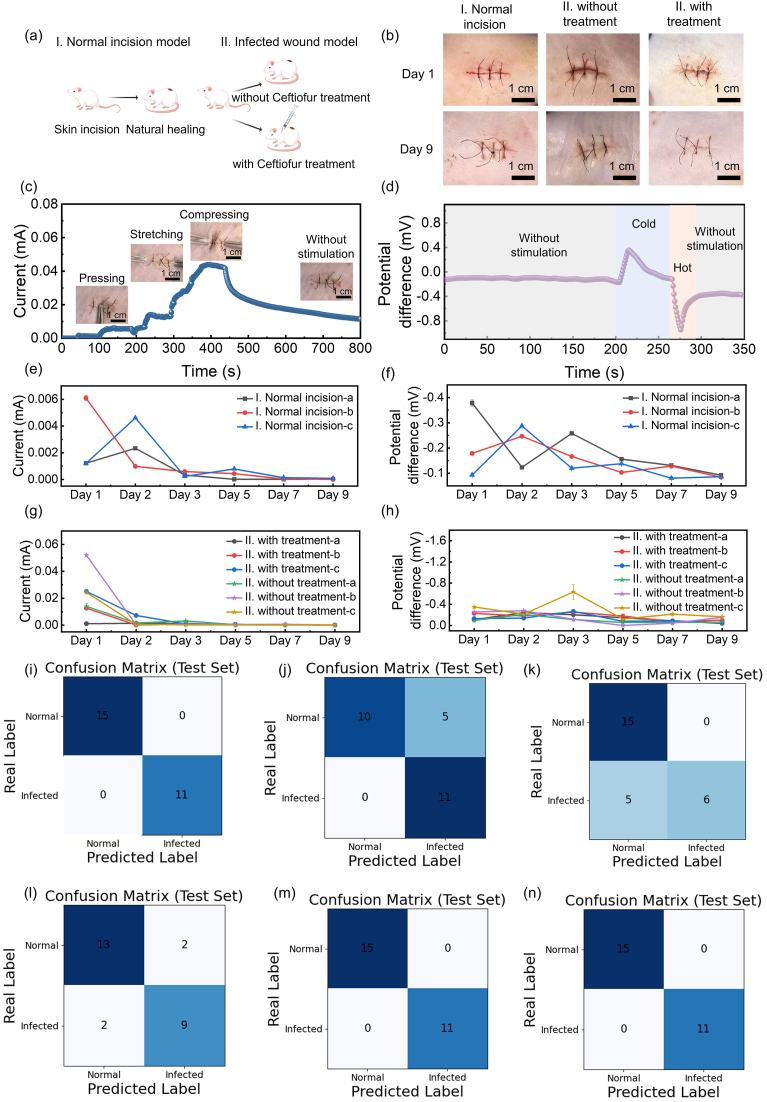
In vivo demonstration of the smart suture. (a) A concept diagram showing the typical changes in inflammation over time for different wound models: normal incision model, infected wound model, and infected wound model treated with Ceftiofur. (b) Representative optical images of three types of wounds on days 1 and 9 after closure with smart suture (n = 3). (c) *I-t* curve of smart suture on wounds under different mechanical stimuli, including pressing, stretching, and compression. (d) Thermoelectric response of smart suture on wounds under hot and cold stimuli. H_2_O_2_ (e) and temperature (f) level variation measured by the smart suture in the normal incision model (n = 3), over a 9-day period. H_2_O_2_ (g) and temperature (h) level variation measured by the smart suture in the infected model, both with (n = 3) and without treatment (n = 3), over a 9-day period. Confusion matrices of the model test results over time. (i–n) Corresponding to days 1, 2, 3, 5, 7, and 9, respectively.

To evaluate the detection capability of the smart suture under different pathological conditions, two wound models, including a normal incision model and an infected incision model, were established. In the infected wound model, wounds treated with ceftiofur were further compared with untreated wounds to distinguish different healing conditions. These models were used to monitor inflammation-related changes at the wound site during the healing process through variations in H_2_O_2_ and temperature signals (Fig. 7a). The smart sutures were fixed at the wound site either by knotting at both ends or using medical ligation clips. As shown in Fig. 7b, the incisions in all three groups were nearly completely healed by day 9. Furthermore, the continuous monitoring capability of the smart suture was evaluated under dynamic mechanical and thermal stimulation conditions. As shown in Fig. 7c, the smart suture exhibited deformation-dependent current responses toward H_2_O_2_ detection under different mechanical stimuli, including pressing, stretching, and compressing, indicating reliable sensing performance under dynamic deformation conditions. In addition, upon applying cooling and heating stimuli to the wound area, the thermoelectric signal exhibited distinct responses and gradually decreased after the stimulation was removed (Fig. 7d). In the normal incision model (Fig. 7e and f), the current response associated with H_2_O_2_ detection decreased significantly by day 3, with only a weak response remaining on day 9. Meanwhile, the local temperature at the wound site showed an obvious downward trend during the healing process, accompanied by a continuous decrease in the thermoelectric voltage signal [84]. In the infected wound model, *Staphylococcus aureus* was inoculated onto the wound site to induce bacterial infection. To suppress bacterial infection and evaluate therapeutic intervention, ceftiofur sodium was subcutaneously injected into the infected wounds every three days. Compared with the normal incision model, the infected wound model exhibited significantly altered electrical responses (Fig. 7g). Meanwhile, the thermoelectric signals showed a more negative shift and larger fluctuations (Fig. 7h), suggesting enhanced inflammatory activity at the wound site. In the treated infected wound group, only slight differences were observed compared with the untreated infected group on day 1, with the treated group exhibiting slightly lower electrochemical and thermoelectric responses. Ceftiofur sodium treatment was initiated after establishment of the infected wound model. Therefore, inflammatory responses initiated during the preceding infection period, including ROS production and local thermal changes, may persist during the early stage after antibacterial intervention. During the subsequent healing stages, both infected groups exhibited gradually decreased signals. The maintained sensing responses throughout the nine-day monitoring period may benefit from the intrinsic coordination stability of the Fe-carboxylate framework in MIL-53(Fe) [85,86]. To further explore the feasibility of multimodal signal integration for wound-state assessment, repeated random training–testing analyses were performed using different random seeds. In each experiment, 80% of the in vivo experimental dataset was used for training, while the remaining 20% was used for testing. Fig. 7i–n summarize representative confusion matrices of the probabilistic classification analysis obtained at different healing stages (days 1, 2, 3, 5, 7, and 9). The corresponding average classification accuracies for these time points were 100%, 81%, 81%, 85%, 100%, and 100%, respectively. Additional evaluation metrics, including precision, recall, and F1-score, are summarized in Table S4. To further evaluate the effectiveness of the proposed classification strategy, the multimodal Naïve Bayes classifier was compared with conventional single-variable dynamic threshold-based classification methods. Although the H_2_O_2_-based threshold classifier exhibited relatively good performance, its decision relied solely on a single biochemical indicator (Figs. S38–55, wound monitoring over Days 1–9). The temperature-based threshold classifier showed inferior predictive capability because temperature changes alone are insufficient to accurately characterize wound inflammation. By integrating both H_2_O_2_ and thermoelectric signals within a probabilistic Bayesian framework, the proposed classifier achieved the best overall classification performance. These results demonstrate that multimodal signal fusion provides more reliable wound-state discrimination than conventional threshold-based analysis using individual sensing modalities. Together, these results suggest the potential utility of the smart suture platform for machine learning-assisted dynamic monitoring of wound-state progression during the healing process. Relatively lower classification accuracies were observed during the intermediate healing stages (days 2–5), which may be associated with less distinguishable physiological differences between wound conditions during these postoperative stages. In contrast, clearer signal differences were observed during the early and late stages, resulting in relatively improved classification performance. These results preliminarily suggest that the combined analysis of electrochemical and thermoelectric signals may provide a useful approach for monitoring dynamic wound-state evolution over time. Nevertheless, we acknowledge that the current machine learning analysis remains a preliminary proof-of-concept evaluation based on a relatively limited in vivo dataset. Therefore, the present framework is intended to demonstrate the feasibility of multimodal signal-assisted wound monitoring rather than establish a generalized clinical prediction model. Future studies with larger datasets and more complex wound conditions will be necessary to further evaluate the robustness and translational potential of the system.

## 3 Conclusion

This work presents the development of a smart suture capable of simultaneously facilitating postoperative wound closure and enabling multimodal monitoring of healing-related signals. The key contribution of this work lies in the device-level integration of a MOF-based electrochemical sensing interface with a thermoelectric fiber in a suturable format, allowing localized detection of complementary biochemical (H_2_O_2_) and biophysical (temperature) signals. A key advantage of the system is the synergistic combination of MOF-based electrochemical sensitivity and thermoelectric fiber-based temperature responsiveness, enabling the smart suture to capture multiple aspects of the wound microenvironment. Beyond enhancing sensing sensitivity, the MOF layer functions as a key sensing interface and contributes to the rapid, stable, and selective multimodal monitoring capability of the smart suture. Exhibiting a high sensitivity of 8450 μA mM^−1^ cm^−2^ and a low detection limit of 77.44 nM for H_2_O_2_, the MOF film enables sensitive detection of inflammation-related oxidative signals. Meanwhile, the TE fiber substrate maintains high thermal sensitivity of 76.6 μV K^−1^, suitable for localized temperature monitoring. Importantly, the fiber-like morphology ensures mechanical integrity, biocompatibility, and flexibility for suturing, while providing improved tissue conformability compared to conventional planar sensors. We have demonstrated the capability of the smart suture to monitor changes in inflammatory levels in wounds under experimental conditions. In addition, a proof-of-concept probabilistic fusion strategy was explored to evaluate the feasibility of jointly modeling multimodal signals, including H_2_O_2_ and temperature responses. Combined with the animal studies, this preliminary machine learning-assisted analysis provides an initial and scalable framework for integrating complex wound-related signals in future intelligent monitoring systems. It should be emphasized that the current platform primarily outputs temporal trends in inflammation-related H_2_O_2_ and temperature signals, which are not equivalent to the clinical gold standard for the diagnosis of wound infection. Therefore, the present system should be regarded as a proof-of-concept tool for dynamic wound-state monitoring rather than an independent diagnostic platform. In future translational studies, the proposed multimodal monitoring strategy could be further optimized by conducting larger-scale animal experiments and refining the machine learning-assisted framework. Collectively, these results provide a proof-of-concept for multimodal sensing in suturable platforms and highlight the potential of such systems for advanced wound monitoring.

## 4 Experimental procedures

### 4.1 Materials and reagents

The thermoelectric (TE) fiber used in this study is a commercially available carbon nanotube (CNT) fiber. CNT utilized in the whole experiment is purchased from ChengduJiacai Technology Co., Ltd. The fiber has a density of 0.5–0.8 g cm^−3^ and an electrical conductivity of (5–7) × 10^4^ S m^−1^. The CNT fiber was used as received without further modification and serves as both the conductive substrate and thermoelectric sensing element. Anhydrous ferric chloride (FeCl_3_) (AR, ≥99.99%), calcium chloride (CaCl_2_) (AR, ≥99.5%), benzene-1,4-dicarboxylic acid (C_8_H_6_O_4_) (AR, ≥99%), N, N-dimethylformamide (DMF, AR, 95%), Nafion (5 wt%), sodium chloride (NaCl) (AR, ≥99.5%), potassium chloride (KCl) (AR, 99.5%), Tris(hydroxymethyl)aminomethane (Tris) (AR, ≥99.5%), and ethanol (AR, ≥99.7%) were both sourced from Aladdin (Shanghai, China). Hydrogen peroxide (30%), D-(+)-Glucose monohydrate (AR, ≥99.7%) was purchased from Sinopharm Chemicals. For electrochemical experiments, the electrolyte solution used was 0.1 M PBS. Deionized (DI) water from Millipore, DirectQ5.

### 4.2 Instrumentation

The surface structures of all products were characterized using a SEM (HITACHI SU8010). TEM images were collected on the FEI-TALOS-F200X TEM. XRD data was acquired using Cu Kα radiation on the Bruker D8 Advance. The chemical composition was characterized by XPS on a Thermo K-Alpha spectrometer. Infrared analysis was performed using the Thermo Nicolet IS5 device. All mechanical tests were performed using a Electrimechanical Universal Testing Machine (UTM 2103). Electrochemical detection performance of H_2_O_2_ was tested using an Autolab N system (Chenhua Instrument, Shanghai, China) employing a three-electrode setup. Stable voltage measurements were conducted with a Keithley 2182A, and precise temperature measurement with thermal imaging was carried out using a Fotric 626C. The ICP-OES test is conducted using Teledyne Leeman Labs (Prodigy Plus). The main instruments used for H&E staining included a tissue processor (Donatello, DIAPATH), an embedding machine (JB-P5, Wuhan Junjie Electronic Co., Ltd.), a paraffin microtome (LEICA RM2235, Leica), a slide warmer/water bath system (PPTHK-21B, Leica), an upright microscope (ECLIPSE Ni, NIKON), and a microscopic image analysis system (DS-Ri2, NIKON).

### 4.3 Deposition of ZnO nanomembrane

Atomic layer deposition (ALD) was performed to coat ZnO nanomembranes on TE fibers within our custom-built chamber at 120 °C, using DEZ and deionized water as precursors. The deposition cycle of the ZnO nanomembrane comprised a 70 ms DEZ pulse, a 6 s wait, a 30 s N_2_ purge, followed by a 70 ms DI water pulse, another 6 s wait, and a final 30 s N_2_ purge.

### 4.4 Preparation of MIL-53(Fe) film on thermoelectric fibers

To synthesize MIL-53(Fe) films on ALD ZnO-coated fibers, the fibers were initially immersed in 100 mL of DMF containing 1.62 g of FeCl_3_ and maintained at 150 °C for 24 h. After cooling to room temperature, 1.66 g of 1,4-dicarboxybenzene (H_2_BDC) was dissolved in DMF and added to the solution, which was then kept at 150 °C for an additional 24 h. The resulting solids were collected by vacuum filtration and dried under vacuum at 60 °C for 12 h, producing uniform MOF layer on the fiber surfaces.

### 4.5 Thermoelectric test

The Seebeck coefficient (α) of the fiber-based samples was measured by applying a controlled temperature gradient across the sample ends using two Peltier elements. An FOTRIC 626C infrared thermal camera was used to determine ΔT of the sample, and ΔV was recorded using a KEITHLEY 2182A nanovoltmeter. Then Seebeck coefficient was calculated by α = ΔV/ΔT.

### 4.6 Electrochemical test

Electrochemical sensing tests were conducted by using Autolab N with a three-electrode configuration. In this setup, an Ag/AgCl reference electrode (in saturated KCl solution) and a graphite rod counter electrode were used, while the smart suture served as the working electrode.

### 4.7 Theoretical simulation

First-principles calculations were carried out on the basis of periodic DFT (Density Functional Theory) using a generalized gradient approximation within the Perdew-Burke-Ernzerhof exchange correction function. Geometry optimization was conducted before energy calculation. The wave functions were constructed by expanding plane waves with an energy cutoff of 450 eV. A Gamma-centered 2 × 2 × 1 k-point mesh was used for the slab models. Convergence criteria for geometry optimization were set to 1.0 × 10^−5^ eV/atom for total energy and 0.05 eV Å^−1^ for forces. To prevent interactions between the two surfaces, a vacuum gap of 15 Å was applied in the periodically repeated slabs. For free energy calculations, entropic corrections and zero-point energy (ZPE) were included. The free energy of species (ΔG) was computed using the standard formula:

ΔG = E + ΔZPE + ΔH – ΔTS

Here, ZPE represents the zero-point energy, ΔH denotes the integrated heat capacity, T is the temperature of the product, and S stands for the entropy.

The electronic stability program (ESP), absorption energy, and partial density of states (PDOS) were calculated using DFT. All DFT calculations were performed with in the DMol^3^ package within Materials Studio. The exchange-correlation energy was computed using the generalized gradient approximation (GGA) with the Perdew-Burke-Ernzerhof (PBE) functional. Grimme's DFT-D3 model was also employed to account for van der Waals interactions. The core electrons were treated using DFT semi-core pseudopotentials.

The absorption energy (ΔE_absorb_) was calculated by the following equation:

ΔE_absorb_ = E_AB_ - E_A_ - E_B_

### 4.8 Animal experiments

Animal experiments were approved by the Science and Technology Ethics Committee of Donghua University (Approval No. DWSY202601270009) and were performed in accordance with the relevant regulations for animal experiments. Healthy adult male rats weighing approximately 300 g were used in this study. During the experiment, rats were first anesthetized with 4–5% isoflurane for induction, followed by maintenance with 1–2% isoflurane in oxygen to ensure an appropriate anesthetic depth throughout the procedure. After anesthesia, the hair on the dorsal skin was removed, and the surgical area was routinely disinfected. A standardized dorsal incision measuring approximately (20 mm) in length was created in each rat. For the infected wound model, 20 μL of a *Staphylococcus aureus* suspension at 1 × 10^8^ CFU mL^−1^, corresponding to approximately 2 × 10^6^ CFU per wound, was applied uniformly to the incision. The inoculated wounds were maintained for more than 24 h to establish infection. For the infected wound with treatment group, after establishment of the infected wound model, ceftiofur sodium was administered by subcutaneous injection at a dose of 0.3 mg per 100 g body weight. Subsequently, the prepared smart sutures were applied to suture the wound sites for real-time H_2_O_2_ and temperature monitoring. In the control group, the wound was directly sutured with the smart suture after incision, without additional bacterial inoculation or therapeutic drug administration.

### 4.9 Data acquisition and feature extraction for bayesian analysis

For machine-learning analysis, both electrochemical and thermoelectric signals were continuously recorded for at least 100 s at each measurement time point. After the signals reached a stable plateau, 20 consecutives paired H_2_O_2_ current and thermoelectric voltage data points were extracted from the steady-state region and used for subsequent Bayesian analysis. Consequently, the classification was performed using stabilized sensing responses rather than instantaneous readings or peak values. This procedure minimizes the influence of transient signal fluctuations and improves the robustness of multimodal signal characterization.

### 4.10 Mechanical deformation test

Mechanical deformation tests were performed to evaluate the mechanical stability of the smart suture. First, the smart suture was bent at different angles of 0°, 40°, 80°, and 120°, and its electrochemical response was measured under each bending condition to assess the effect of bending deformation on sensing performance. Subsequently, the smart suture was subjected to repeated bending cycles, and electrochemical measurements were conducted after 0, 50, 100, and 150 bending cycles to evaluate its signal retention capability under cyclic mechanical deformation. Finally, the smart suture was repeatedly rubbed on the surface of 2000-grit sandpaper for 0, 20, 40, and 60 cycles, and the electrochemical responses before and after abrasion were recorded to assess its stability under mechanical wear conditions.

### 4.11 Electrochemical performance in different biological media

Electrochemical performance in different biological media was evaluated to assess the stability of the smart suture under biologically relevant conditions. The smart suture was immersed in simulated body fluid (SBF, Shanghai Yuanye), fetal bovine serum (FBS, Gibco), 10% FBS solution, and heat-inactivated FBS, respectively. Heat-inactivated FBS was prepared by incubating FBS in a water bath at 56 °C for 30 min. Subsequently, the smart sutures treated with different media were subjected to electrochemical measurements to evaluate their sensing stability in complex biological environments.

## CRediT authorship contribution statement

**Yang Xiao:** Data curation, Formal analysis, Writing – original draft, Writing – review & editing. **Xingyu Gao:** Data curation. **Tingting Sun:** Conceptualization, Writing – review & editing. **Jiasheng Jiang:** Data curation, Methodology, Writing – review & editing. **Jing Lu:** Data curation, Investigation. **Chengbin Zhu:** Data curation. **Jiaxi Zhang:** Data curation, Formal analysis. **Yang Sun:** Data curation. **Yu Zhang:** Data curation. **Xinyi Ke:** Data curation. **Gaoshan Huang:** Conceptualization, Writing – review & editing. **Shengda Tian:** Formal analysis. **Xue Ke:** Investigation. **Jiajun Li:** Investigation. **Zhe Zhao:** Conceptualization, Funding acquisition, Writing – review & editing. **Xuanyong Liu:** Funding acquisition.

## Ethics approval and consent to participate

Animal experiments were approved by the Science and Technology Ethics Committee of Donghua University (Approval No. DWSY202601270009) and were performed in accordance with the relevant regulations for animal experiments.

## Declaration of competing interest

Xuanyong Liu is an editorial board member for Bioactive Materials and was not involved in the editorial review or the decision to publish this article. All authors declare that there are no competing interests.
