# Supplementary material for: Multimodal smart suture for dynamic postoperative wound monitoring
Source: Bioact Mater. 2026 Jul 30;66:1186–201. doi: 10.1016/j.bioactmat.2026.07.044 (PMC13451855; doi:10.1016/j.bioactmat.2026.07.044)
Supplement: Supplementary file 1 [file mmc1.docx]

**Supporting Information**

**Multimodal Smart Suture for Dynamic Postoperative Wound Monitoring**

**Table S1. Summary of H_2_O_2_ sensing performances of MOF-based structures reported in literature published in recent years.**

| MOF Composite | Sensitivity (μA mM^−1^ cm^−2^) | Linear Range (μM) | LOD (μM) | RSD | Ref. |
| --- | --- | --- | --- | --- | --- |
| LIGE-PB | / | 1-200 | 260 | 3.2% | [1] |
| Na, O-*g*-C_3_N_4_ | 17.57 | 1-50 | 0.1 | 2.89% | [2] |
| CS/GOP | 0.77 | 0.5-200 | 17.3 | / | [3] |
| AuNPs/porous GaN | 281.5 | 10-100 | 2 | 2.93% | [4] |
| Smart suture | 8450 | 0.5-200 | 0.077 | 2.23% | This work |

**Table S2. Summary of the comparison of temperature response time and Seebeck coefficient reported in recent years.**

| Thermoelectric  material | Seebeck coefficient (μV K^-1^) | Temperature response time (ms) | Ref. |
| --- | --- | --- | --- |
| MFSOTE | 35.5 | 2000 | [5] |
| MXene-AgNW/PEDOT:PSS-TeNW | 25.3 | 1800 | [6] |
| PEDOT/MWCNT | 13.8 | 470 | [7] |
| c-SWCNT/PDA/Glu | 48.5 | 1500 | [8] |
| MOF/CNT | 46.1 | 950 | [9] |
| PCL-SWCNT | 40.8 | 1600 | [10] |
| Smart suture | 76.6 | 514 | This work |

**Table S3. Summary of the sensing performances of fiber-based structures reported in recent years.**

| Materials | Analyte | Sensitivity | Linear Range | LOD | Stability | Site | Ref. |
| --- | --- | --- | --- | --- | --- | --- | --- |
| CoFe-DAzyme | Ascorbic Acid | / | 0.1-100 μM | 0.075 μM | 7 days | Plant | [11] |
| Sodium ionophore X | Na^+^ | 52.8 mV decade^−1^ | 130 μM-200 mM | 18.27 μM | 7 days | Brain | [12] |
| Lactate oxidase | Lactate | / | 0-28 mM | 150 μM | 12 h | Epidermis | [13] |
| Cu_1_/C_3_N_4_ | H_2_O_2_ | 5.45 nA 10^−6^ M^−1^ | / | 5 μM | 7 days | Brain | [14] |
| Smart suture | H_2_O_2_ | 8450 μA mM^−1^ cm^−2^ | 0.5-200 μM | 0.077 μM | 9 days | Epidermis | This work |
|  | temperature | 76.6 μV K^-1^ | / | / |  |  |  |

**Table S4. Classification statistics based on machine learning.**

| Day | Wound model | Precision | recall | f1-score | Accuracy |
| --- | --- | --- | --- | --- | --- |
| 1 | Infected | 1.00 | 1.00 | 1.00 | 1 |
|  | Normal | 1.00 | 1.00 | 1.00 |  |
| 2 | Infected | 0.69 | 1.00 | 0.81 | 0.81 |
|  | Normal | 1.00 | 0.67 | 0.80 |  |
| 3 | Infected | 1.00 | 0.55 | 0.71 | 0.81 |
|  | Normal | 0.75 | 1.00 | 0.86 |  |
| 5 | Infected | 0.82 | 0.82 | 0.82 | 0.85 |
|  | Normal | 0.87 | 0.87 | 0.87 |  |
| 7 | Infected | 1.00 | 1.00 | 1.00 | 1 |
|  | Normal | 1.00 | 1.00 | 1.00 |  |
| 9 | Infected | 1.00 | 1.00 | 1.00 | 1 |
|  | Normal | 1.00 | 1.00 | 1.00 |  |

**Figure S1**. System-level integration strategy for multimodal postoperative wound monitoring.

**Figure S2.** SEM images of smart suture with low magnifications.

**Figure S3.** SEM images of smart suture with high magnifications.

**Figure S4.** EDS mapping images of smart suture.

**Figure S5.** EDS spectrum of smart suture, and the elemental contents.

**Figure S6.** I-V curve of the smart suture and TE fiber.

**Figure S7.** Optical microscope images of the TE fiber.

**Figure S8.** Optical microscope images of the ALD-TE fiber.

**Figure S9.** Optical microscope images of the smart suture.

**Figure S10.** Pore size distribution of (a) TE fiber (b) ALD-TE fiber and (c) smart suture.

**Figure S11.** Stress-strain curves of TE fiber, ALD-TE fiber, and smart suture.

**Figure S12.** To assess suture-tissue interaction, the required drag force normalized by circumference was recorded for commercial PET, silk fiber, TE fiber, and the smart suture in porcine meat (n = 3).

**Figure S13.** Stress-strain curves of smart suture, silk, and PET fiber.

**Figure S14.** SEM images of the smart suture before and after ultrasonic treatment.

**Figure S15.** CV curves of the smart suture in electrolytes with and without H_2_O_2_ (1 mM).

**Figure S16.** *I-t* curves of smart suture with successive addition of 1 mM H_2_O_2_ in 0.1M PBS at different potentials.

**Figure S17.** The response currents of smart suture and thermosensitive fiber for 0.1 mM H_2_O_2_ in 0.1 M PBS at 1.2 V.

**Figure S18.** *I-t* curve of smart suture in 0.1 M PBS with successive addition of 1 mM H_2_O_2_, 0.1 mM NaCl, 0.1 mM KCl, 0.1 mM CaCl_2_, 0.1 mM Tris, 0.1 mM Glu, and 1 mM H_2_O_2_ at 1.2 V.

**Figure S19.** *I-t* curves of the smart suture in PBS and BSA-containing PBS with 1mM H_2_O_2_. The fiber was stabilized in the corresponding solution for 5 min.

**Figure S20.** *I-t* curves of the smart suture with successive additions of 1 mM H_2_O_2_ in SBF (a), FBS (b), 10% FBS (c), and 10% heat-inactivated FBS (d).

**Figure S21.** *I-t* curves of smart suture when 1 mM of H_2_O_2_ is added for 5 times.

**Figure S22.** Batch-to-batch reproducibility of the smart suture at the same H_2_O_2_ concentration of 1 mM.

**Figure S23.** *I-t* curves of the smart suture toward successive additions of H_2_O_2_ with randomly varied concentrations. H_2_O_2_ was sequentially added at concentrations of 0.5 μM, 80 μM, 0.5 μM, and 80 μM.

**Figure S24.** *I-t* curves of smart suture in 0.1 M PBS with 1mM H_2_O_2_, and the pH values are set to be 5, 6, 7, and 8.

**Figure S25.** Photograph of the smart suture wrapped around a plastic tube, with an inset

showing it supporting a 20 g mass.

**Figure S26.** Electrochemical stability of the smart suture under mechanical deformation. (a) Electrochemical response of the smart suture under bending angles from 0−120°. (b) Electrochemical response after 0−150 bending cycles. (c) Electrochemical response after 0−60 friction cycles.

**Figure S27.** (a) ΔT and corresponding thermovoltage of the TE fiber, and (b)linear fitting of thermovoltage with ΔT.

**Figure S28.** (a)ΔT and corresponding thermovoltage of the ALD-TE fiber, and (b) linear fitting of thermovoltage with ΔT.

**Figure S29.** (a) ΔT and corresponding thermovoltage of the smart suture, and (b) linear fitting of thermovoltage with ΔT.

**Figure S30.** The response time of smart suture to temperature sensing (514 ms).

**Figure S31.** Time-dependent thermoelectric response of smart suture under stepwise temperature gradients.

**Figure S32.** High magnification SEM image (a), low magnification SEM image (b), and corresponding EDS elemental mapping (c) of the smart suture.

**Figure S33.** High magnification SEM image (a), low magnification SEM image (b), and corresponding EDS elemental mapping (c) of the smart suture after 30 repeated penetrations through fresh porcine skin.

**Figure S34.** Electrochemical response toward H_2_O_2_ (a) and thermoelectric response to temperature (b) of the smart suture before and after 30 repeated penetrations through fresh porcine skin.

**Figure S35.** The sensing process involves the reaction of H_2_O_2_ on the surface of TE fiber (side view).

**Figure S36.** The sensing process involves the reaction of H_2_O_2_ on the surface of TE fiber (top view).

**Figure S37.** The cumulative release of Fe over a period of 7 days in control, commercial, and smart suture.

Figure S38. Performance evaluation of the Bayesian classifier based on H_2_O_2_ sensing signals (day 1). (a) Classification performance metrics as a function of the decision threshold on the training set, with the optimal threshold indicated by the dashed line. (b) ROC curves for the training and testing datasets. (c) Distribution of H_2_O_2_ sensing responses for the normal and infected groups, with the selected decision threshold indicated by the dashed line. (d) Confusion matrix of the training dataset. (e) Confusion matrix of the testing dataset. (f) Comparison of classification performance metrics.

Figure S39. Performance evaluation of the Bayesian classifier based on temperature sensing signals (day 1). (a) Classification performance as a function of the decision threshold on the training set, with the optimal threshold indicated by the dashed line. (b) ROC curves for the training and testing datasets. (c) Distribution of temperature sensing responses for the normal and infected groups, with the selected decision threshold indicated by the dashed line. (d) Confusion matrix of the training dataset. (e) Confusion matrix of the testing dataset. (f) Comparison of classification performance metrics.

Figure S40. Comparison between the Naïve Bayes classifier and single-variable dynamic threshold classification methods (day 1). Confusion matrices of the Naïve Bayes classifier for the training (a) and testing (b) datasets. ROC curves (c).

Figure S41. Performance evaluation of the Bayesian classifier based on H_2_O_2_ sensing signals (day 2). (a) Classification performance metrics as a function of the decision threshold on the training set, with the optimal threshold indicated by the dashed line. (b) ROC curves for the training and testing datasets. (c) Distribution of H_2_O_2_ sensing responses for the normal and infected groups, with the selected decision threshold indicated by the dashed line. (d) Confusion matrix of the training dataset. (e) Confusion matrix of the testing dataset. (f) Comparison of classification performance metrics.

Figure S42. Performance evaluation of the Bayesian classifier based on temperature sensing signals (day 2). (a) Classification performance as a function of the decision threshold on the training set, with the optimal threshold indicated by the dashed line. (b) ROC curves for the training and testing datasets. (c) Distribution of temperature sensing responses for the normal and infected groups, with the selected decision threshold indicated by the dashed line. (d) Confusion matrix of the training dataset. (e) Confusion matrix of the testing dataset. (f) Comparison of classification performance metrics.

Figure S43. Comparison between the Naïve Bayes classifier and single-variable dynamic threshold classification methods (day 2). Confusion matrices of the Naïve Bayes classifier for the training (a) and testing (b) datasets. ROC curves (c).

Figure S44. Performance evaluation of the Bayesian classifier based on H_2_O_2_ sensing signals (day 3). (a) Classification performance metrics as a function of the decision threshold on the training set, with the optimal threshold indicated by the dashed line. (b) ROC curves for the training and testing datasets. (c) Distribution of H_2_O_2_ sensing responses for the normal and infected groups, with the selected decision threshold indicated by the dashed line. (d) Confusion matrix of the training dataset. (e) Confusion matrix of the testing dataset. (f) Comparison of classification performance metrics.

Figure S45. Performance evaluation of the Bayesian classifier based on temperature sensing signals (day 3). (a) Classification performance as a function of the decision threshold on the training set, with the optimal threshold indicated by the dashed line. (b) ROC curves for the training and testing datasets. (c) Distribution of temperature sensing responses for the normal and infected groups, with the selected decision threshold indicated by the dashed line. (d) Confusion matrix of the training dataset. (e) Confusion matrix of the testing dataset. (f) Comparison of classification performance metrics.

Figure S46. Comparison between the Naïve Bayes classifier and single-variable dynamic threshold classification methods (day 3). Confusion matrices of the Naïve Bayes classifier for the training (a) and testing (b) datasets. ROC curves (c).

Figure S47. Performance evaluation of the Bayesian classifier based on H_2_O_2_ sensing signals (day 5). (a) Classification performance metrics as a function of the decision threshold on the training set, with the optimal threshold indicated by the dashed line. (b) ROC curves for the training and testing datasets. (c) Distribution of H_2_O_2_ sensing responses for the normal and infected groups, with the selected decision threshold indicated by the dashed line. (d) Confusion matrix of the training dataset. (e) Confusion matrix of the testing dataset. (f) Comparison of classification performance metrics.

Figure S48. Performance evaluation of the Bayesian classifier based on temperature sensing signals (day 5). (a) Classification performance as a function of the decision threshold on the training set, with the optimal threshold indicated by the dashed line. (b) ROC curves for the training and testing datasets. (c) Distribution of temperature sensing responses for the normal and infected groups, with the selected decision threshold indicated by the dashed line. (d) Confusion matrix of the training dataset. (e) Confusion matrix of the testing dataset. (f) Comparison of classification performance metrics.

Figure S49. Comparison between the Naïve Bayes classifier and single-variable dynamic threshold classification methods (day 5). Confusion matrices of the Naïve Bayes classifier for the training (a) and testing (b) datasets. ROC curves (c).

Figure S50. Performance evaluation of the Bayesian classifier based on H_2_O_2_ sensing signals (day 7). (a) Classification performance metrics as a function of the decision threshold on the training set, with the optimal threshold indicated by the dashed line. (b) ROC curves for the training and testing datasets. (c) Distribution of H_2_O_2_ sensing responses for the normal and infected groups, with the selected decision threshold indicated by the dashed line. (d) Confusion matrix of the training dataset. (e) Confusion matrix of the testing dataset. (f) Comparison of classification performance metrics.

Figure S51. Performance evaluation of the Bayesian classifier based on temperature sensing signals (day 7). (a) Classification performance as a function of the decision threshold on the training set, with the optimal threshold indicated by the dashed line. (b) ROC curves for the training and testing datasets. (c) Distribution of temperature sensing responses for the normal and infected groups, with the selected decision threshold indicated by the dashed line. (d) Confusion matrix of the training dataset. (e) Confusion matrix of the testing dataset. (f) Comparison of classification performance metrics.

Figure S52. Comparison between the Naïve Bayes classifier and single-variable dynamic threshold classification methods (day 7). Confusion matrices of the Naïve Bayes classifier for the training (a) and testing (b) datasets. ROC curves (c).

Figure S53. Performance evaluation of the Bayesian classifier based on H_2_O_2_ sensing signals (day 9). (a) Classification performance metrics as a function of the decision threshold on the training set, with the optimal threshold indicated by the dashed line. (b) ROC curves for the training and testing datasets. (c) Distribution of H_2_O_2_ sensing responses for the normal and infected groups, with the selected decision threshold indicated by the dashed line. (d) Confusion matrix of the training dataset. (e) Confusion matrix of the testing dataset. (f) Comparison of classification performance metrics.

Figure S54. Performance evaluation of the Bayesian classifier based on temperature sensing signals (day 9). (a) Classification performance as a function of the decision threshold on the training set, with the optimal threshold indicated by the dashed line. (b) ROC curves for the training and testing datasets. (c) Distribution of temperature sensing responses for the normal and infected groups, with the selected decision threshold indicated by the dashed line. (d) Confusion matrix of the training dataset. (e) Confusion matrix of the testing dataset. (f) Comparison of classification performance metrics.

Figure S55. Comparison between the Naïve Bayes classifier and single-variable dynamic threshold classification methods (day 9). Confusion matrices of the Naïve Bayes classifier for the training (a) and testing (b) datasets. ROC curves (c).

References

[1] T. A. Matias, L. V. de Faria, R. G. Rocha, M. N. T. Silva, E. Nossol, E. M. Richter, R. A. A. Muñoz. Prussian blue-modified laser-induced graphene platforms for detection of hydrogen peroxide, Microchim Acta. 189 (5) (2022) 188, <https://doi.org/10.1007/s00604-022-05295-5>.

[2] A. Mohammad, M. E. Khan, T. Yoon, M. Hwan Cho. Na,o-co-doped-graphitic-carbon nitride (na,o-g-c_3_n_4_) for nonenzymatic electrochemical sensing of hydrogen peroxide, Appl Surf Sci. 525 (2020) 146353, <https://doi.org/10.1016/j.apsusc.2020.146353>.

[3] A. Nasir, S. Khalid, A. Mazare, T. Yasin. Non-enzymatic hydrogen peroxide detection on a novel nanohybrid composite of chitosan and grafted graphene oxide, Mater Res Bull. 167 (2023) 112427, <https://doi.org/10.1016/j.materresbull.2023.112427>.

[4] M.-R. Zhang, X.-Q. Chen, G.-B. Pan. Electrosynthesis of gold nanoparticles/porous gan electrode for non-enzymatic hydrogen peroxide detection, Sensor Actuat B-Chem. 240 (2017) 142-147, <https://doi.org/10.1016/j.snb.2016.08.124>.

[5] F. Zhang, Y. Zang, D. Huang, C.-a. Di, D. Zhu. Flexible and self-powered temperature–pressure dual-parameter sensors using microstructure-frame-supported organic thermoelectric materials, Nat Commun. 6 (1) (2015) 8356, <https://doi.org/10.1038/ncomms9356>.

[6] F. Li, Y. Liu, X. Shi, H. Li, C. Wang, Q. Zhang, R. Ma, J. Liang. Printable and stretchable temperature-strain dual-sensing nanocomposite with high sensitivity and perfect stimulus discriminability, Nano Lett. 20 (8) (2020) 6176-6184, <https://doi.org/10.1021/acs.nanolett.0c02519>.

[7] Y. Cui, X. He, W. Liu, S. Zhu, M. Zhou, Q. Wang. Highly stretchable, sensitive, and multifunctional thermoelectric fabric for synergistic-sensing systems of human signal monitoring, Adv Fiber Mater. 6 (1) (2024) 170-180, <https://doi.org/10.1007/s42765-023-00339-8>.

[8] B. Huo, S. Liu, C. Du, F. Kuang, Y. Lv, X. Wu, P. Liu, L. Liang, G. Chen, C.-Y. Guo. Organic carbonization modification of carbon nanotubes with stable thermoelectric performance at high temperature, Chem Eng J. 506 (2025) 160213, <https://doi.org/10.1016/j.cej.2025.160213>.

[9] M.-H. Lin, C.-H. Hsu, D.-Y. Kang, C.-L. Liu. Correlating framework structures and thermoelectric performance of metal–organic framework/carbon nanotube thermoelectric hybrids with n–p type inversion, Chem Eng J. 485 (2024) 149732, <https://doi.org/10.1016/j.cej.2024.149732>.

[10] S. Liu, H. Li, H. Wang, J. Kong, J. J. Koh, C. He. High thermoelectric performance carbon nanotube/polycaprolactone composites for sustainable energy harvesting, Small. 21 (44) (2025) e06983, <https://doi.org/10.1002/smll.202506983>.

[11] J. Zhu, J. Xia, X. Liu, X. Yang, Y. Pan, C. Kang, X. Chang, J. Zhao, Y. Zhao, J. Ping. Implantable fiber sensor for continuous monitoring of light-regulated ascorbic acid dynamics, ACS Nano. 20 (15) (2026) 11624-11636, <https://doi.org/10.1021/acsnano.5c18174>.

[12] F. Zhao, Y. Liu, H. Dong, S. Feng, G. Shi, L. Lin, Y. Tian. An electrochemophysiological microarray for real-time monitoring and quantification of multiple ions in the brain of a freely moving rat, Angew Chem, Int Ed. 59 (26) (2020) 10426-10430, <https://doi.org/10.1002/anie.202002417>.

[13] F. Tehrani, H. Teymourian, B. Wuerstle, J. Kavner, R. Patel, A. Furmidge, R. Aghavali, H. Hosseini-Toudeshki, C. Brown, F. Zhang, K. Mahato, Z. Li, A. Barfidokht, L. Yin, P. Warren, N. Huang, Z. Patel, P. P. Mercier, J. Wang. An integrated wearable microneedle array for the continuous monitoring of multiple biomarkers in interstitial fluid, Nat Biomed Eng. 6 (11) (2022) 1214-1224, <https://doi.org/10.1038/s41551-022-00887-1>.

[14] X. Gao, W. Ma, J. Mao, C.-T. He, W. Ji, Z. Chen, W. Chen, W. Wu, P. Yu, L. Mao. A single-atom cu–n2 catalyst eliminates oxygen interference for electrochemical sensing of hydrogen peroxide in a living animal brain, Chem Sci. 12 (45) (2021) 15045-15053, <https://doi.org/10.1039/D1SC04755H>.
